# Hypertensive disorders in pregnancy and risk of asthma in the offspring: a systematic review and meta-analysis

**DOI:** 10.3389/fmed.2026.1739697

**Published:** 2026-07-28

**Authors:** Nancy D. Calzada, Carlos A. Barba, Carlos Quispe-Vicuña, Raul H. Sandoval-Ato, Christian A. Rodriguez-Saldaña, Judith Yangali-Vicente, Oriana Rivera-Lozada, Joshuan J. Barboza

**Affiliations:** 1 Universidad de Huánuco, Huánuco, Peru; 2 Hospital d'Olot i comarcal de la Garrotxa, Olot, Spain; 3 Grupo de Investigación NEMECS: Neurociencias, Metabolismo, Efectividad Clínica y Sanitaria, Universidad Científica del Sur, Lima, Peru; 4 Escuela de Medicina Humana, Universidad Antenor Orrego, Piura, Peru; 5 Hospital de la Amistad Perú Corea Santa Rosa II-1, Piura, Peru; 6 Facultad de Responsabilidad Social, Universidad Anahuac, Mexico City, Mexico; 7 Vicerrectorado de Investigación, Universidad Señor de Sipán, Chiclayo, Peru; 8 Escuela de Medicina, Universidad Señor de Sipán, Chiclayo, Peru

**Keywords:** childhood asthma, gestational hypertension, hypertensive disorders of pregnancy, meta-analysis, preeclampsia, systematic review

## Abstract

**Background:**

Hypertensive disorders of pregnancy (HDP), including preeclampsia, gestational hypertension, and eclampsia, are common pregnancy complications that may have long-term consequences for offspring health. Emerging evidence suggests a potential link between maternal hypertension and childhood asthma, though findings remain inconsistent. This systematic review and meta-analysis aimed to synthesize the existing evidence on the association between HDP and asthma risk in offspring.

**Methods:**

A comprehensive search of PubMed, Scopus, Web of Science, and EMBASE was conducted from database inception to 3 December 2024, restricted to peer-reviewed published studies. The eligibility criteria followed the PICOS framework. Observational studies (cohort, case–control, cross-sectional) assessing the association between HDP and asthma in offspring were included. Effect measures were converted to odds ratios (OR) with 95% confidence intervals (CI), and a meta-analysis was performed using random- and fixed-effects models. Heterogeneity was assessed jointly with Cochran’s *Q* test (*p* < 0.10) and the *I*^2^ statistic, and publication bias was examined through funnel plots and Egger’s test.

**Results:**

Twenty studies encompassing over three million mother–infant pairs were included. The pooled OR for asthma risk in offspring of mothers with HDP was 1.14 (95% CI: 1.07–1.21), indicating a significant association. Subgroup analyses revealed a positive association with both preeclampsia (OR: 1.20; 95% CI: 1.12–1.28) and gestational hypertension (OR: 1.18; 95% CI: 1.02–1.36). Heterogeneity was high in some subgroups, such as gestational hypertension (*I*^2^ = 92.6%; *p* < 0.0001), suggesting variability across studies. As a secondary outcome, the association between HDP and wheezing in the offspring was assessed. The pooled OR was 1.07 (95% CI: 0.99–1.17), not reaching statistical significance, although positive trends were observed in some subtypes.

**Conclusion:**

Maternal HDP is associated with an approximately 14–20% increased risk of asthma in offspring, highlighting the need for targeted early-life monitoring and intervention strategies. Further research is warranted to elucidate underlying mechanisms and optimize maternal–fetal health outcomes.

**Systematic review registration:**

IS: CRD420251000229.

## Introduction

Hypertensive disorders of pregnancy (HDP), including preeclampsia, gestational hypertension, and eclampsia, are significant medical complications affecting approximately 5–10% of pregnancies worldwide (1). These conditions not only pose an immediate risk to maternal and fetal health but have also been associated with long-term adverse outcomes in offspring, including an increased risk of chronic diseases (2). Among these, childhood asthma has emerged as an area of growing interest, given that intrauterine exposure to factors such as placental hypoxia, systemic inflammation, and oxidative stress might alter lung and immune system development, predisposing children to develop respiratory diseases (3).

The existing evidence on the association between HDP and asthma risk in offspring is heterogeneous and sometimes contradictory. Some studies suggest a positive association (3, 4), while others find no significant link (5, 6). This inconsistency may be attributed to differences in study designs, populations assessed, exposure and outcome definitions, and methods of adjustment for confounding factors. Despite the clinical and public health relevance of this potential association, a comprehensive and up-to-date synthesis of the available evidence to allow robust conclusions to be drawn has not been conducted.

An updated search conducted in October 2024 in PROSPERO, MEDLINE, the Cochrane Database of Systematic Reviews, and JBI Evidence Synthesis did not identify any ongoing systematic reviews addressing this specific question with a current and methodologically robust approach. Although previous reviews, such as those by Conlan (7) and Li (8), have examined the association between HDP and the risk of asthma in offspring, they present methodological limitations that justify an updated synthesis. Conlan included only nine studies up to April 2020 and did not adequately disaggregate HDP subtypes or distinguish between outcomes such as asthma and wheezing, despite evaluating the certainty of the evidence. Li, although more recent, combined studies with different clinical outcomes (asthma and wheezing) into a single meta-analysis and did not conduct subgroup analyses by HDP subtype or outcome (a detailed comparison of the present review with previous systematic reviews and meta-analyses is provided in Supplementary Table 5). In this context, the present systematic review and meta-analysis aims to fill this gap by incorporating 20 primary studies, distinguishing between specific HDP subtypes and respiratory outcomes, and applying rigorous methodological tools—including subgroup analyses and formal assessment of the certainty of evidence—to generate a more accurate and clinically relevant synthesis. This review will provide a solid foundation for future research and public health policies aimed at mitigating the impact of HDP on childhood respiratory health.

To ensure clarity and international relevance, key terms are defined as follows. Hypertensive disorders of pregnancy (HDP): preeclampsia, gestational hypertension and eclampsia, according to the standard definitions of the International Society for the Study of Hypertension in Pregnancy (ISSHP, 2018). Childhood asthma: diagnosis of asthma in children under 18 years of age, confirmed by clinical criteria or lung function testing, according to international guidelines [Global Initiative for Asthma (GINA), 2022].

The objective of this review is to assess the association between hypertensive disorders during pregnancy and the risk of asthma in offspring, synthesizing the available evidence from observational studies.

## Methods

This study was a systematic review and meta-analysis conducted following the Preferred Reporting Items for Systematic Reviews and Meta-Analyses (PRISMA) 2020 guidelines (Supplementary Table 1) (9). The protocol was registered in PROSPERO (registration number: CRD420251000229).

### Search strategy

The eligibility criteria of this review were structured according to the PICOS framework: Population—pregnant women and their offspring from birth to 18 years of age; Intervention/Exposure—maternal hypertensive disorders of pregnancy (HDP), including preeclampsia, gestational hypertension, eclampsia, and chronic hypertension with or without superimposed preeclampsia; Comparator—pregnant women without HDP; Outcomes—incidence or prevalence of asthma in the offspring (primary), and recurrent wheezing, asthma treatment, and lung function parameters (secondary); Study design—observational studies (cohort, case–control, and cross-sectional). A comprehensive electronic search restricted to peer-reviewed published studies was performed in PubMed, Scopus, Web of Science, and EMBASE, from database inception to 3 December 2024. An initial limited search in MEDLINE (PubMed) was used to identify keywords and index terms in titles and abstracts, which were then employed to refine the complete search strategy adapted for each database. Reference lists of included studies and relevant systematic reviews were also screened. No language restrictions were applied. Reviews, editorials, letters, conference abstracts, commentaries, case reports, study protocols without primary results, and unpublished material (including grey literature and dissertations) were not eligible. The full search syntax for each database is provided in Supplementary Table 2.

### Eligibility criteria

Studies eligible for inclusion were observational in design, including cohort, case–control, and cross-sectional studies. No time restrictions were applied. The studies needed to evaluate the association between hypertensive disorders in pregnancy (HDP)—including preeclampsia, gestational hypertension, chronic hypertension, preeclampsia superimposed on chronic hypertension, and HELLP syndrome—and the risk of asthma in offspring. The review included studies that examined pregnant individuals diagnosed with HDP, as defined by established guidelines such as those from the International Society for the Study of Hypertension in Pregnancy (ISSHP) and the American College of Obstetricians and Gynecologists (ACOG). Studies that evaluated asthma or respiratory symptoms such as recurrent wheezing in offspring as the primary outcome, regardless of the diagnostic method used, were eligible. Offspring from birth to adolescence (up to 18 years) were included as participants in asthma outcomes. Comparator groups consisted of pregnant individuals without HDP. Both singleton and multiple pregnancies were included, provided the data were reported separately or could be extracted independently.

Studies that exclusively focused on individuals with pre-existing chronic hypertension were excluded due to differences in underlying mechanisms and outcomes. Studies that failed to define or differentiate HDP from other hypertensive conditions were also excluded. Offspring with congenital anomalies or genetic syndromes that could independently affect respiratory outcomes were excluded as well. Narrative and systematic reviews, meta-analyses, editorials, letters to the editor, commentaries, case reports, conference abstracts and proceedings, study protocols without primary results, and unpublished reports were also excluded. Studies retrieved only as conference abstracts or for which a full peer-reviewed manuscript was not available were not eligible.

### Exposures and comparators

The exposure of interest was pregnant individuals diagnosed with HDP. The comparator group consisted of pregnant individuals without HDP.

### Outcomes

The primary outcome was the incidence of asthma in offspring. Secondary outcomes included recurrent wheezing, asthma treatment, and lung function parameters such as forced expiratory volume in 1 s (FEV1), forced vital capacity (FVC), and the FEV1/FVC ratio. Asthma incidence and prevalence were determined based on validated physician diagnoses, standardized survey reports, medical records, or health databases. Wheezing was defined as a whistling sound during breathing, assessed through caregiver reports, medical records, and validated questionnaires. Recurrent wheezing was defined as at least two episodes after age two or the repeated use of asthma medication. Asthma treatment was determined by the use of inhaled corticosteroids, nebulizers, leukotriene modifiers, or oral corticosteroids, measured through physician prescriptions and documented medication use. Following ATS/ERS standards, spirometry was used to measure lung function parameters.

### Study selection and data extraction

Following the search, all identified citations were collated and uploaded into EndNote 21/2023 (Clarivate Analytics, PA, USA), where duplicates were removed. A pilot screening was conducted before two independent reviewers screened the titles and abstracts against the inclusion criteria. Potentially relevant studies were retrieved in full, and their citations were imported into the JBI System for the Unified Management, Assessment, and Review of Information (JBI SUMARI; JBI, Adelaide, Australia). Two reviewers independently assessed the full-text articles, and any disagreements were resolved through discussion or consultation with a third reviewer. Reasons for exclusion were documented and reported in the final review. The selection process was reported following the PRISMA flow diagram (9).

### Risk of bias assessment

The methodological quality of eligible studies was independently assessed by two reviewers using the Newcastle–Ottawa Scale (NOS) (10), which has been previously applied in systematic reviews and meta-analyses on pregnancy-induced hypertension and related outcomes (11). Any discrepancies were resolved through discussion or by consulting a third reviewer. Studies were classified as having a low, moderate, or high risk of bias based on criteria related to selection, comparability, and outcomes (for cohort studies) or selection, comparability, and exposure (for case–control studies). The overall quality assessment was determined from the total number of stars awarded per study; according to the authors’ criteria, studies with scores ≤ 6 were considered to be at high risk of bias (low quality), and scores > 6 were considered to be at low risk of bias (high quality). Assessments were performed by two authors and conflicts were resolved by a third author (JJB).

### GRADE assessment

The certainty of evidence for all reported outcomes was assessed by consensus using the Grading of Recommendations, Assessment, Development, and Evaluations (GRADE) approach (12). Factors considered included risk of bias, inconsistency, indirectness, imprecision, and publication bias. The certainty of evidence was categorized as high, moderate, low, or very low, and results were summarized in Summary of Findings (SoF) tables using GRADEpro GDT (Supplementary Table 3).

### Data synthesis

To perform the meta-analysis, we first extracted the measures of association reported in each study, prioritizing odds ratios (OR) and their respective 95% confidence intervals (CI). We sought to select the adjusted ORs reported by the studies and, if a study presented more than one fitted model, the model with the most covariates was selected. In cases where the OR was not reported directly but data were available in a contingency table, it was calculated manually using the formula (a × d)/(b × c), where a and b represent cases and controls in the exposed group, and c and d in the unexposed group. Additionally, studies reporting risk ratios (RR), hazard ratios (HR), or incidence rate ratios (IRR) were converted to OR using established approximations: OR = RR/(1 − P0 + (P0 × RR)) and OR = HR/(1 − P0 + (P0 × HR)), where P0 represents the baseline prevalence of the outcome in the unexposed group. All estimates were transformed to their logarithmic scale using log(OR) = ln(OR), and the standard error was calculated with SElog(OR) = (ln(upper CI) − ln(lower CI))/3.92. Once these values were obtained, a meta-analysis was performed using fixed- and random-effects models. The fixed-effects model assumed a common between-study variance and weighted individual estimates by the inverse of the variance of each log(OR). In contrast, the random-effects model used the DerSimonian and Laird method to estimate between-study heterogeneity by calculating *τ*^2^. Statistical heterogeneity across studies was assessed using Cochran’s *Q* test and the *I*^2^ statistic. A *p* value < 0.10 for the *Q* test and/or an *I*^2^ > 50% were considered indicative of substantial heterogeneity, with *I*^2^ values of 25, 50 and 75% interpreted as low, moderate, and high heterogeneity, respectively (13). Both metrics (*I*^2^ and *p* value) were reported jointly for every pooled estimate and subgroup analysis. To explore possible sources of variability, subgroup analyses were performed according to the type of hypertensive disorder, offspring age, and other population characteristics. In addition to the planned subgroup analyses by HDP subtype, three pre-specified subgroup analyses were performed for the primary outcome to explore potential sources of heterogeneity: (a) by continent of the source population (Europe, North America, Asia–Pacific, and Oceania); (b) by exposed-group sample size (≤ 500 vs. > 500 mother–infant pairs); and (c) by year of publication (≤ 2015 vs. > 2015). The number of additional subgroup analyses was deliberately restricted to avoid spurious findings. Sensitivity analyses excluded studies with a high risk of bias or incomplete data. Publication bias was assessed through visual inspection of the funnel plot and statistical tests (Egger’s and Begg’s tests), applying the trim-and-fill method if significant asymmetry was detected. Finally, results were interpreted considering the magnitude of the effect, its clinical relevance, and the potential methodological limitations of the included studies.

## Results

### Selection of studies

**Figure 1:**
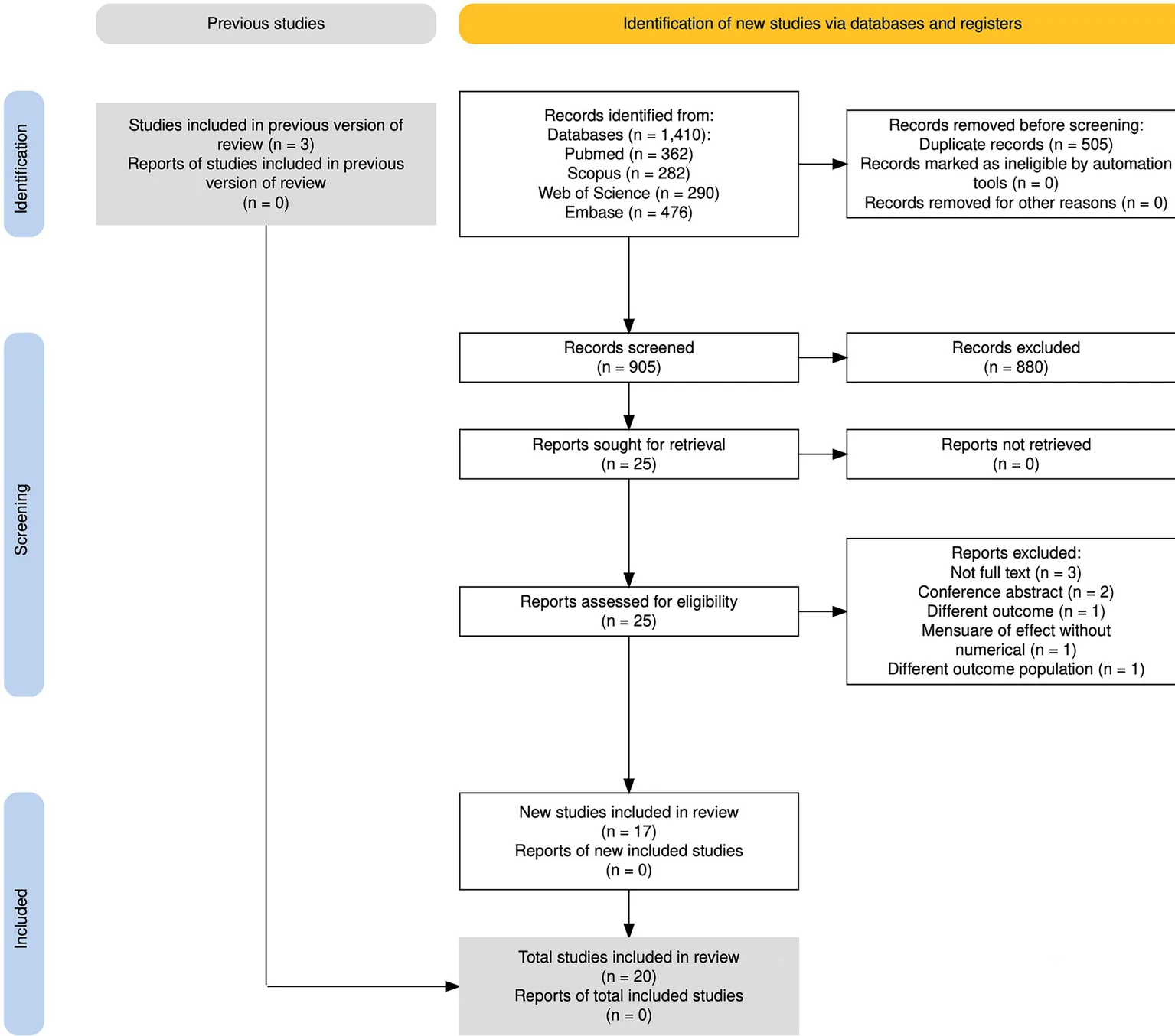
PRISMA 2020 flow diagram of study identification, screening, eligibility, and inclusion.

A total of 1,410 records were identified through database searches, including 362 from PubMed, 282 from Scopus, 290 from Web of Science, and 476 from Embase. After removing 505 duplicate records, 905 unique records remained for screening. Following the initial screening, 880 records were excluded based on their titles and abstracts. Consequently, 25 reports were sought for full-text retrieval, all successfully obtained. These 25 reports were assessed for eligibility, leading to the exclusion of seven studies for various reasons, including three not available as full texts, two conference abstracts, one with a different outcome, one without numerical measures of effect, and one involving a different population. Ultimately, 17 new studies were included in the systematic review. In total, 20 studies were incorporated into the final analysis, including three from the previous version of the review and 17 newly identified studies (Figure 1). The excluded studies and their justification are listed in Supplementary Table 4.

### Characteristics of included studies

The included studies encompassed various epidemiological and methodological characteristics, covering diverse populations, study designs, and data sources. The studies were conducted across multiple countries, including Australia, the United States, the United Kingdom, Sweden, Norway, Denmark, China, Japan, and the Netherlands, with study designs primarily consisting of cohort studies (both prospective and retrospective) and register-based analyses. Data sources varied significantly, with some studies utilizing national birth registries, hospital discharge databases, electronic medical records, prescription drug databases, and longitudinal cohort studies that followed participants from birth through adolescence or even adulthood. The duration of follow-up ranged from a few months to over 30 years, with most studies tracking children for at least five years to assess asthma development (Table 1).

**Table 1:** Characteristics of included studies (epidemiological features).

Author	Year	Country	Study design	Data source	Duration of follow-up	Exposure factor	Number of patients with hypertensive disorders in pregnancy	Number of patients without hypertensive disorders in pregnancy	Number of patients with asthma	Number of patients without asthma
Algert et al. (23)		Australia	Cohort prospective	Admitted Patient Data Collection, Midwives Data Collection, ABS mortality data	3 years	Any maternal hypertension	23,992	782	7,245	240,511
Arroyo et al. (25)	2023	Boston	Cohort	Massachusetts General Hospital Maternal–Child Cohort (MMCC)	From birth to 5.9 years	Preeclampsia	624	14,305	92	532
						HDP	1,139	13,790	178	961
Aspberg et al. (16)	2010	Sweden	Cohort	Swedish Medical Birth Register, Swedish Prescribed Drug Register, Swedish Hospital Discharge Register	15 years	Preeclampsia	41,389	1,296,930	2,466	38,923
Byberg et al. (20)	2014	Norway	historically matched cohort	Stavanger study: Medical Birth Registry of Norway, registros hospitalarios y cuestionarios ISAAC	10.8 years (women) 11.8 years (male)	Pre-eclmapsia: Mild/moderate	214	366	506	80
					10.8 years (women) 11.8 years (male)	Pre-eclmapsia: Severe	214	366	506	80
					12.8 years	Pre-eclmapsia: Mild/moderate	169	279	388	198
					12.8 years	Pre-eclmapsia: Severe	169	279	388	198
Byberg et al. (14)	2022	Sweden	Cohort	Registros nacionales suecos (MBR, NPR, PDR, MGR, LISA).	8 years	Pre-eclmapsia: Mild/moderate	22,233	757,885	28,852	750,859
						Pre-eclmapsia: Severe	22,233	757,885	42,884	736,827
						Pre-eclmapsia: Mild/moderate	22,233	757,885	14,036	765,675
						Pre-eclmapsia: Severe	22,233	757,885	14,812	764,899
			Cohort		6 years	Pre-eclmapsia: Mild/moderate	6,821	235,296	257	4,389
						Pre-eclmapsia: Severe	6,821	235,296	147	2,236
Henderson and Quenby (21)	2021	United Kingdom	Cohort	Millennium cohort study (MCS)	5 years	Gestational hypertension	950	11,500	1857	10,593
Kelly et al.	2022	United Kingdom	Cohort	Millennium cohort study (MCS)	7 years	HDP	986	12,075	2,151	10,910
Liu et al. (17)	2015	Denmark	Register-based cohort study with nested case–control and case-sibling analyses.	Danish national registries (Danish Medical Birth Registry, Danish National Patient Register, and Danish National Prescription Registry).	From 3 to 18 years, or until asthma diagnosis, emigration, death, or end of study (2010).	Preeclampsia	3,402	112,120	115,522	808,011
Ma et al. (4)	2023	China	Retrospective Cohort.	Physical Health Monitoring Project of Primary and High School Students in Guangzhou (PHMPPHSS).	12 years	Gestational hypertesnion	5,330	161,442	19,722	147,050
Magnus et al. (15)	2016	Norway	Cohort prospective	Information from the Medical Birth Registry of Norway (MBRN), the Norwegian Prescription Database (NorPD) and the Norwegian Education Database.	7 years	Preeclampsia	Registry linkage: 16329	Registry linkage: 390578	Registry linkage: 19938	Registry likage: 386969
						Preeclampsia	MoBa: 1747	MoBa: 43281	MoBa: 2882	MoBa: 42146
Mckeever et al. (26)	2012	United Kingdom	Cohort prospective	General Practice Research Database, UK	6 months	Eclampsia	NR	NR	2,671	NR
Mirzakhani et al. (18)	2019	United States	Cohort prospective	Vitamin D Antenatal Asthma ReductionTrial (IVDAART)	3 years	Preeclampsia	67	739	125	681
Mirzakhani et al. (27)	2019	United States	Cohort prospective	Used the data from Vitamin D Antenatal Asthma Reduction Trial (VDAART)	6 years	Preeclampsia	61	646	79	628
Nafstad et al. (6)	2003	Norway	Retrospective cohort study	Medical Birth Registry of Norway (MBRN) and National Insurance Administration Register (NIAR)	29 years (1967–1996)	Preeclampsia	98,188	1,428,341	5,938	1,520,591
Nafstad et al. (28)	2000	Norway	Prospective cohort	Oslo Birth Cohort	4 years	Pregnancy complications (hyperemesis, hypertension, and preeclampsia)	141	2,382	164	2,367
Shaheen et al. (5)	2016	United Kingdom	Cohort study	Avon Longitudinal Study of Parents and Children (ALSPAC)	8,5 years	Preeclampsia	1,675	9,268	1,655	6,440
						Gestational hypertension	238	9,268	2,963	7,815
						Hypertension before pregnancy	420	9,268	879	7,285
				
Stokholm et al. (24)	2017	Denmark	Prospective cohort study	Copenhagen Prospective Studies on Asthma in Childhood (COPSAC2000)	7 years	Preeclampsia	23	388	47	289
				Danish national registries	35 years		62,728	1,635,910	90,114	NR
Wilmink et al. (19)	2018	The Netherlands	Prospective cohort study	Generation R Study, Erasmus Medical Center, Rotterdam	From pregnancy to 10 years postnatal	Gestational hypertension	206	4,868	313	4,581
						PE/HELLP	91	4,803	313	4,581
Yang et al. (22)	2021	Japan	Prospective cohort study	Japan Environment and Children’s Study (JECS)	10 years	Hypertensive disorders in pregnancy	2,334	75,171	NR	NR
						Maternal Hypertension	3,333	75,171	NR	NR
Yang et al. (29)	2023	Taiwan	Cohort	Taiwan National Health Insurance Research Database (NHIRD) and Maternal and Child Health Database (MCHD).	16 years	Gestational Hypertension	3,041	12,164	180	554
						Preeclampsia or eclampsia	3,041	12,164	180	554

The exposure of interest was hypertensive disorders in pregnancy (HDP), including preeclampsia, gestational hypertension, chronic hypertension, and eclampsia. Some studies further categorized preeclampsia as mild, moderate, or severe to assess the potential dose–response relationship with asthma risk in offspring. The number of participants varied widely among studies, with sample sizes ranging from small, highly controlled cohorts of fewer than 1,000 participants to extensive nationwide registry studies that included over a million individuals. The number of children diagnosed with asthma also varied significantly, with some studies reporting fewer than 100 cases while others included tens of thousands of asthma cases. Comparator groups consisted of children born to mothers without hypertensive disorders in pregnancy, allowing for a direct assessment of the impact of HDP on asthma outcomes (Table 1).

In terms of methodological characteristics, asthma diagnosis was assessed through various approaches, including physician-diagnosed asthma recorded in medical registries, self-reported parental questionnaires based on standardized protocols such as the International Study of Asthma and Allergies in Childhood (ISAAC), and prescription records of asthma medications. Some studies used specific ICD-10 codes for asthma identification to ensure diagnostic consistency, while others relied on survey-based definitions of wheezing and recurrent asthma symptoms. Inclusion criteria varied between studies, most requiring live singleton births, adequate follow-up data, and detailed maternal health records. Exclusion criteria commonly included multiple pregnancies, preexisting maternal chronic hypertension, congenital anomalies, low birth weight, and missing data on hypertensive disorders or asthma diagnosis (Table 2).

**Table 2:** Characteristics of included studies (methodological features).

Author	Year	Pregnancy year (media DS, median and/or range)	Age of offspring at asthma diagnosis (years)	Evaluation method of with Hypertensive disorders in pregnancy	Evaluation method of with asthma	Inclusion criteria	Exclusion criteria	Outcomes	Confounding factors	Statistical methods
Algert et al. (23)	2011	NR	3–5 years	Maternal hospital admission records for hypertension (Hospital discharge or birth record summaries)	ICD-10 codes (J45 or J46) in hospital discharge summaries (age 3–5)	NR	NR	Asthma	Parity, socioeconomic, maternal asthma and male child, prematurity, transient tachypnea, respiratory distress syndrome, neonatal sepsis and jaundice/phototherapy.	Logistic regression; crude and adjusted relative risks (RRs) and odds ratios (aORs)
Arroyo et al. (25)	2023	NR	5 years	Definition according to American College of Obstetricians and Gynecologists (blood pressure, delivery records, preeclampsia criteria)	Primary diagnosis recorded in electronic medical record or asthma medication events within a 12-month period	Mother and child treated at a MGB facility Child with at least one medical record before turning one year old and another between 3 and 5.9 years old.	Initial prenatal visit after 20 weeks Multiple pregnancies Preexisting chronic hypertension - Children with congenital diseases or respiratory abnormalities, immunodeficiency or congenital heart disease.	Asthma	Maternal age, BMI, smoking, maternal asthma history, health insurance type, marital status, birth weight, gestational age, sex of child, and route of delivery.	Logistic regression models adjusted for maternal and child factors.
Aspberg et al. (16)	2010	NR	Until age 15	Medical records	Medical records: Anti-asthma medication prescription and hospital discharge records	Singletons born in Sweden between 1990 and 2003; alive in 2005	Multiple births; non-live births; under 2 years of age	Asthma	Year of birth, maternal age, parity, smoking, and involuntary childlessness, prematurity, Apgar, Apgar <7 at 5 min, Neonatal sepsis and/or pneumonia, Neonatal mechanical ventilation, Apgar <7 at 5 min, Neonatal sepsis and/or pneumonia, Neonatal jaundice, neonatal phototherapy	Mantel–Haenszel adjusted odds ratio (OR)
Byberg et al. (20)	2014	NR	10.8 years (women) 11.8 years (male)	Clinical definition: blood pressure ≥90 mmHg (diastolic), proteinuria ≥2 + after 20 weeks	FU1: Maternal-reported (physician-diagnosed)	Live births in Stavanger 1993–1995; Longitudinal follow-up with questionnaires and biological samples	No specific data, but excludes missing or unfit cases for longitudinal follow-up.	Asthma Current asthma	gender, birth weight z-score adjusted for gestational age, being firstborn, Cesarean section, maternal smoking during pregnancy, gestational age in weeks, maternal age, maternal body mass index (kg/m2) and maternal asthma	Multiple logistic regression models adjusted for relevant covariates
			10.8 years (women) 11.8 years (male)	Proteinuria with dipstick ≥ + 3 and diastolic blood pressure of ≥110 mmHg						
			12.8 years	Clinical definition: blood pressure ≥90 mmHg (diastolic), proteinuria ≥2 + after 20 weeks	FU2: Self- reported (ISAAC)			FV1% FVC FV1%/FVC	gender, birth weight z-score adjusted for gestational age, being firstborn, Cesarean section, maternal age, maternal smoking during pregnancy, gestational age in weeks, maternal body mass index (kg/m2) and maternal asthma.	
			12.8 years	Proteinuria with dipstick ≥ + 3 and diastolic blood pressure of ≥110 mmHg						
Byberg et al. (14)	2022	NR	0–2 years	CIE-10 basado en registros médicos. Preeclampsia leve a moderada” (O14.0), “Preeclampsia no especificada” (O14.9) y “Preeclampsia con hipertensión preexistente” (O11.9). “Preeclampsia grave” (O14.1), “Hemólisis, enzimas hepáticas elevadas y recuento bajo de plaquetas (HELLP)” (O14.2) y “Eclampsia” (O15.0, O O15.2 y O15.9).	Registered medical diagnoses and medications.	Singletons and live births, and survived beyond the first day of life, complete data	NR	Asthma onset	age, smoking, Swedish oral snuff, education, asthma, birth country, body mass index; child: gender, firstborn; and family disposable income.	Logistic and Cox regression as HR
			0–2 years							
			>2 years							
			>2 years							
			6 years					Prevalent non-allergic asthma	age, smoking, Swedish oral snuff, education, asthma, birth country, body mass index; child: gender, firstborn; and family disposable income	Logistic and Cox regression as OR
			6 years					Prevalent allergic asthma	age, smoking, Swedish oral snuff, education, asthma, birth country, body mass index; child: gender, firstborn; and family disposable income	
Henderson and Quenby (21)	2021	-	5 years	Self-report of gestational hypertension (PIH) or preeclampsia (PET) through MCS questionnaires.	Based on self-reports from the ISAAC questionnaire.	Participation in the first three waves of the Millennium Cohort Study (MCS) Biological mothers after singleton pregnancy	Multiple pregnancies. Surveys not answered by the biological mother.	Asma Wheezing	Age at delivery, Parity Smoking during pregnancy History of essential, hypertension prior to pregnancy, Household income quintile, Child’s sex, Gestational and sex-adjusted birth weight, Gestational age at birth, Type of delivery	logistic regression model
Kelly et al.	2022	NR	7 years	based on self-reports	Based on self-reports from the ISAAC questionnaire.	Children born between 2000 and 2002, follow-up to age 7 years, complete data on HDP and asthma, and complete surveys completed by biological mother.	Multiple pregnancies, non-biological mothers, incomplete data on HDP or asthma, lack of response in waves.	Asthma Wheezing Regular use of asthma medication	Maternal age, ethnicity, education, maternal asthma history, smoking during pregnancy, BMI, poverty, birth weight, parity, and sex of the child.	Multivariate logistic regression, causal mediation analysis, stratified analysis.
Liu et al. (17)	2015	NR	3 Years	Diagnosis based on ICD-8 and ICD-10 codes recorded in Danish hospitals.	At least one hospitalization for asthma or two prescriptions for anti-asthma medications (specific ICD-10 and ATC codes).	Live births in Denmark between 1993 and 2007 with complete data on gestational age, birth weight, and follow-up to 3 years or more.	Multiple births, missing or unlikely pregnancy/weight data, death or emigration before age 3 years.	Asthma	Maternal age, parity, social status, maternal smoking, maternal asthma, child gender, and year of birth.	Conditional logistic regression, nested case–control analysis, case-sibling analysis.
Ma et al. (4)	2023	NR	6 years	Based on medical records, according to international standards, hypertension is defined as ≥140/90 mmHg on two separate occasions after 20 weeks of gestation.	Confirmed medical diagnosis of asthma reported by parents through a questionnaire based on the ISAAC protocol.	Children aged 6–12 years who participated in the PHMPPHSS in 2017–2018, with complete data on gestational complications and asthma diagnosis.	Multiple births, mothers with pre-existing hypertension or diabetes, incomplete data on gestational complications or asthma diagnosis.	Asthma	Child sex, region of residence, age, maternal pre-pregnancy body mass index, maternal educational level, family income, smoking during pregnancy.	Binomial logistic regression
Magnus et al. (15)	2016	NR	7 years	Medical Birth Registry of Norway, considered preeclampsia (HELLP syndrome, eclampsia, early preeclampsia, mild preeclampsia and severe preeclampsia).	At age 7 years if the child had been prescribed at least one asthma medication in the past 12 months, plus at least one second prescription filled within 12 months of the first.	NR	NR	Asthma	maternal age, parity, education, child’s sex, rmaternal smoking during pregnancy	Log-binomial regression models, relative risks (RR) and 95% IC, Mediation analysis using logistic decomposition, conventional multivariate adjustment, conditional logistic regression analysis
		NR	7 years		If the mother reported in the questionnaire that the child had been diagnosed with asthma by a doctor and also had current symptoms or had used asthma medications in the past 12 months.	NR	NR	Asthma	Maternal age, parity, education, asthma, child’ssex, maternal smoking during pregnancy and pre-pregnancybody mass index.	
Mckeever et al. (26)	2012	NR	Mean: 2.20 years	Oxford Medical Information System and Read codes	Oxford Medical Information System, which was derived from International Classification of Disease, version 8,	Antibiotics used during pregnancy (by trimester): penicillin, amoxycillin, coamoxiclav, macrolides, cephalsporin, and other antibiotics, paternal exposure to antibiotics during pregnancy, abortion (therapeutic or spontaneous/miscarriages) and stillbirths before the birth of the index child, number of medical visits recorded in 6-month intervals from birth.	Visits for vaccinations and allergic disease outcomes.	Asthma	Gender, parental allergic disease, parental smoking, mater nal age, general practice, antibiotics in the first 6 months of life, child’s consulting behavior in the first 6 months of life in quartiles, and year of birth.	Cox regression models, hazard ratios (HR), 95% confidence intervals (CI) and multivariate analyses, using robust standard errors.
Mirzakhani et al. (18)	2019	26.7 (5.40) 18.0–38.80	3 years	pautas del Congreso Estadounidense de Obstetras y Ginecólogos (ACOG)	parental report of physician’s diagnosis of asthma on the prospective study questionnaires	Enrolled pregnant women who were randomized according to the intention-to-treat paradigm and their living offspring with follow-ups for the trial’s primary and secondary outcomes “recurrent asthma or wheezing” at age 3 years and “preeclampsia.” Women between 18 and 39 years with estimated gestational ages of 10 and 18 weeks (singleton fetus), non-smokers, with a history of asthma or physician-diagnosed atopy, or a partner (biological father of the child) with a history of asthma or physician-diagnosed atopy. Eligible participants were assessed according to the VDAART protocol and enrolled if eligible	Multiple gestacion Smoked or udes drugs	Asthma recurrent wheeze	These maternal variables included: trial arm (placebo vs. vitamin D supplementation), parity (first child or more), maternal age, race (African American, white, and other), body mass index (mg/kg2) at first appointment, gestational age at delivery, gestational diabetes, mode of delivery, child sex, child birth weight, study center, annual household income, maternal marital and educational status, maternal partner’s asthma status, and plasma 25-hydroxyvitamin D at enrollment (ng/ml). Total 25-hydroxyvitamin D levels were measured using a chemiluminescence assay on the DiaSorin Liaison machine at the data coordinating center.	Student’s t test, chi-square, Fisher exact test, Weibull regression models, Crude and adjusted hazard ratios (HR and aHR) were calculated with 95% confidence intervals (CI)
Mirzakhani et al. (27)	2019	NR	6 years	NR	Asthma was defined as a maternal orcaregiver report of physician-diagnosed asthma.	Eligible participants in the VDAART were non-smoker adult women (18–39 years old) with gestational ages of 10–18 weeks; with history of asthma or atopy, or whose partner (biologic father of the child) had a history of asthma or atopy and with intent to participate for at least 4 years.	NR	Asthma	NR	Logistic regression, multivariable logistic regression models (*p* for trend <0.0001), Mantel–Haenszel chi square
Nafstad et al. (6)	2003	NR	7.5 -- 25.5 years	Medical records (ICD-8)	Derived from NIAR Diagnosis verified by a pediatrician or lung physician; defined by ICD-7 and ICD-9	Live births in Norway between 1967 and 1993, registered in the Medical Birth Registry of Norway (MBRN), Linked to the National Insurance Administration Register (NIAR).	Children who died or emigrated before 5 months of age; missing data on birth weight, parity, or maternal age	Asthma	Gender, maternal age, maternal education, birth order, year of birth, plurality, single-parent family, maternal atopy, diabetes mellitus, lower respiratory tract infections, other maternal infections during pregnancy, and geographical district of birth maternal atopy, diabetes mellitus, lower respiratory tract infections, and other maternal infections during pregnancy. and other pregnancy complications	Logistic regression, cox regression
Nafstad et al. (28)	2000	NR	4 years	Questionnaire administered at the maternity wards soon after birth	Medical diagnosis confirmed by questionnaires at 4 years.	Live birth children >2000 g, without serious illnesses, Norwegian families, resident in Oslo.	Birth weight <2000 g, severe illness, non-Norwegian language, families with drug abuse	Asthma	Genetic propensity and other pregnancy and socioeconomic factors and an episode of early life lower respiratory tract infection	Multivariable logistic regression, confounder-adjusted models.
Shaheen et al. (5)	2016	NR	7.5 years	Based on International Society for the Study of Hypertension in Pregnancy (ISSHP) criteria, using blood pressure and proteinuria measurements from obstetric records	Maternal report of doctor-diagnosed asthma at 7.5 years	Mothers and offspring from the ALSPAC cohort with available data on hypertensive disorders of pregnancy and respiratory outcomes	Mothers with incomplete data, those with missing information on hypertensive disorders or childhood outcomes	Asthma	Offspring sex, maternal asthma, maternal pre-pregnancy BMI, maternal age, maternal parity, maternal education, multiple pregnancy, maternal ethnicity, maternal smoking during pregnancy	Logistic regression for binary outcomes (wheezing, asthma, atopy), linear regression for continuous lung function outcomes (FEV1, FVC, FEF25-75), four stages of adjustment including confounders
			18 months					Wheeze at 18 m		
			7 yeras					Wheeze at 7 years		
			8 years					Lung function (FEV1, FVC)		
Stokholm et al. (24)	2017	With preeclampsia: 31.6 (4.6)	7 years	Personal interview and registry validation	Clinical investigator-diagnosed asthma based on predefined criteria	Children born to mothers with doctor-diagnosed asthma, excluding preterm births (<36 weeks), severe congenital abnormalities, or early lung symptoms.	Gestational age <36 weeks, severe congenital abnormalities, lung symptoms before enrollment.	Asma	Mode of delivery	COPSAC2000: Chi-square test, Student’s t-test, logistic regression, generalized estimating equation (GEE) models, generalized linear models.
		NR	To up 35 years	ICD-8 and ICD-10 codes in hospital records	and ICD-10 codes in hospital records	Live-born children in Denmark (1978–2012) whose mothers were born after 1952.	NR	Inhaled corticosteroids treatment FEV1	Age and calendar year, birth weight, gestational age, sex, mode of deivery, parity, multiple births, season of birth, maternal age, andmother’sdisease.	Registry-based cohort: Log-linear Poisson regression models, incidence rate ratios (IRR), population attributable risk (PAR) fractions.
Wilmink et al. (19)	2018	NR	10 years	Validated oscillometric digital device (OMRON HEM-907), ISSHP & ACOG criteria	Spirometry (ATS/ERS) + parental report	Singleton pregnancies with complete prenatal BP data & postnatal follow-up at 10 years	Multiple gestations, missing HDP or asthma outcome data	Asma	Maternal age, ethnicity, pre-pregnancy BMI, education, parity, smoking, folic acid use, child’s sex	Multivariable logistic regression, mediation analysis, conditional regression
								Sibilancia	Maternal age, ethnicity, pre-pregnancy BMI, education, parity, smoking, folic acid use, child’s sex	
								Lung function (FEV1, FVC, FEV1/FVC)	maternal age, maternal ethnicity, pre-pregnancy body mass index, educational level, nulli parity, smoking habits during pregnancy, folic acid use and child’s sex	
Yang et al. (22)	2021	NR	3 years	Medical records and guideline of Japanese Society for Study of HDP Japan.	International Study of Asthma and Allergies in Childhood (ISAAC)	Maternal systolic and diastolic BPs (SBP, DBP) in early (14 weeks), mid- (14–28 weeks) and late (28 weeks) pregnancy	Children who, by 3 years of age, had been lost to follow-up, or for whom data on allergic diseases was missing.	Asma	Maternal age, maternal education level, maternal BMI, maternal history of pregnancy abnormality, maternal allergy history, maternal smoking, maternal drinking, paternal smoking and education level, family income, pet keeping, parity and sex	Logistic regression model and multiple imputation
		NR	3 years	Medical records maternal blood pressure at early (≤14 weeks), mid (14–28 weeks), and late (≥28 weeks) pregnancy. Hypertension (SBP ≥ 140 and/or DBP ≥ 90 mm Hg)				Sibilancua	Maternal age, maternal education level, maternal BMI, maternal history of pregnancy abnormality, maternal allergy history, maternal smoking, maternal drinking, paternal smoking, paternal education level, family income, pet keeping, parity and sex.	
Yang et al. (29)	2023	NR	NR	ICD-9-CM and ICD-10-CM (diagnosis of gestational hypertension).	ICD-9-CM and ICD-10-CM (minimum 3 outpatient visits or 1 hospitalization).	Primiparous women, diagnosis of gestational hypertension, 4:1 matched controls.	Mothers with chronic hypertension prior to pregnancy.	Asthma	NR	Cox proportional hazards regression model, Kaplan–Meier method
				NR	NR	NR	NR		NR	

The statistical approaches employed to analyze the association between HDP and asthma varied, with studies utilizing logistic regression, Cox proportional hazards models, and generalized estimating equations. Most studies adjusted for key confounders such as maternal age, pre-pregnancy BMI, smoking during pregnancy, parity, socioeconomic status, birth weight, gestational age, mode of delivery, and family history of asthma. Effect measures included odds ratios (OR), risk ratios (RR), hazard ratios (HR), and incidence rate ratios (IRR), with corresponding 95% CI (Table 3).

**Table 3:** Characteristics of included studies (statistical features).

Outcome	Years	Definition of exposition	Years patients with asthma	*n* = with asthma	*n* = whitout asthma	Type of measure of effect	OR/RR/HR	ICi	ICs
Preeclampsia/Asthma									
Arroyo et al. (25)	2023	General (new-onset hypertension after 20 weeks’ gestation)	5 years	92	532	OR	0.95	0.74	1.20
Aspberg et al. (16)	2010	General	< 15 years	2,466	961	OR	1.24	1.19	1.29
Byberg et al. (20)	2014	Mild/moderate	10.8 years (women) 11.8 years (male)	506	80	OR	0.87	0.38	1.97
Byberg et al. (20)	2014	Severe pre-eclampsia	10.8 years (women) 11.8 years (male)	506	80	OR	0.72	0.19	2.77
Byberg et al. (20)	2014	Mild/moderate	12.8 years	388	198	OR	1.11	0.45	2.73
Byberg et al. (20)	2014	Severe pre-eclampsia	12.8 years	388	198	OR	0.69	0.15	3.18
Byberg et al. (14)	2022	Mild/moderate	0–2 years	28,852	750,859	HR	1.13	01.05	1.21
Byberg et al. (14)	2022	Severe pre-eclampsia	0–2 years	42,884	736,827	HR	1.78	1.64	1.95
Byberg et al. (14)	2022	Mild/moderate	>2 years	14,036	765,675	HR	1.11	1.00	1.24
Byberg et al. (14)	2022	Severe pre-eclampsia	>2 years	14,812	764,899	HR	1.26	01.08	1.46
Byberg et al. (14)	2022	Mild/moderate	6 years	257	4,389	OR	1.17	1.00	1.36
Byberg et al. (14)	2022	Severe pre-eclampsia	6 years	147	2,236	OR	1.51	1.23	1.84
Liu et al. (17)	2015	General (International Classification of Diseases, 8th revision)	3–18 years	3,402	27,365	IRR	1.19	1.15	1.24
Magnus et al. (15)	2016	Medical Birth Registry of Norway (síndrome HELLP, eclampsia, preeclampsia temprana, preeclampsia leve; y preeclampsia grave)	7 years	19,938	386,969	RR	1.29	1.19	1.39
Magnus et al. (15)	2016	Medical Birth Registry of Norway (síndrome HELLP, eclampsia, preeclampsia temprana, preeclampsia leve; y preeclampsia grave)	7 years	2,882	42,146	RR	1.19	0.99	1.44
Mirzakhani et al. (18)	2019	General (American Congress of Obstetricians and Gynecologists guidelines)	3 years	125	681	HR	1.62	01.02	2.57
Mirzakhani et al. (27)	2019	General	6 years	79	628	RR	1.71	1,1	2.70
Nafstad et al. (6)	2003	General (ICD-8)	<27	5,938	1,520,591	OR	01.08	0.97	1.19
Shaheen et al. (5)	2016	General (La hipertensión gestacional se definió como presión arterial sistólica ≥ 140 mmHg y/o presión arterial diastólica ≥ 90 mmHg en dos ocasiones después de 20 semanas de gestación, con proteinuria de al menos 1+)	7 years	1,655	6,440	OR	1.23	0.80	1.88
Stokholm et al. (24)	2017	General (ICD-8 ICD-10)	until age 7 years	47	289	OR	1.13	0.31	4.13
Stokholm et al. (24)	2017	General (ICD-8 ICD-10)	until age 35 years	90,114	NR	IRR	01.09	01.05	1.12
Eclampsia/Asthma									
McKeever et al. (26)	2012	Oxford Medical Information System and Read	2.20	2,671	21,871	HR	1.16	0.84	1.59
Preeclampsia/Allergic asthma									
Byberg et al. (14)	2022	Mild/moderate	6 years	119	4,251	OR	0.91	0.72	1.15
Byberg et al. (14)	2022	Severe pre-eclampsia	6 years	77	2,166	OR	1.31	0.98	1.74
HDP/Asthma									
Arroyo et al. (25)	2023		5 years	178		OR	1.10	0.92	1.31
Kelly et al.	2022	“Presión arterial elevada, eclampsia/preeclampsia o toxemia	7 years	205	1946	OR	1.35	1.15	1.59
Yang et al. (22)	2021	Medical record review	3 years	NR	NR	OR	0.98	0.85	1.13
Preclampasia-Eclampsia/Asthma									
Yang et al. (29)	2023	General (ICD-8 ICD-10)	NR	NR	NR	HR	0.85	0.65	1.12
Wilmink et al. (19)	2018	Validated oscillometric digital device (OMRON HEM-907), ISSHP & ACOG criteria	10 years	313	4,581	OR	0.80	0.32	02.01
Gestational hypertension/Asthma									
Henderson and Quenby (21)	2021	Self-reported history of HYP or PET, which was combined in the Millennium Cohort Study (MCS) first wave questionnaire. Gestational hypertension “high blood pressure, eclampsia/preeclampsia, or toxemia,”	5 years	1857	10,593	OR	1.20	1.00	1.45
Ma et al. (4)	2023	Gestational hypertension (defined as blood pressure ⩾140 mmHg systolic or ⩾90 mmHg diastolic on two separate occasions at least 4 h apart after 20 weeks of pregnancy when previous blood pressure was normal)	12 years	19,722	147,050	OR	1.48	1.37	1.59
Shaheen et al. (5)	2016	General (La hipertensión gestacional se definió como presión arterial sistólica ≥ 140 mmHg y/o presión arterial diastólica ≥ 90 mmHg en dos ocasiones después de 20 semanas de gestación)	7 years	2,963	7,815	OR	01.05	0.88	1.25
Wilmink et al. (19)	2018	Validated oscillometric digital device (OMRON HEM-907), ISSHP & ACOG criteria	10 years	313	4,581	OR	0.99	0.57	1.73
Yang et al. (29)	2023	General (ICD-8 ICD-10)	3 years	NR	NR	HR	1.12	01.02	1.23
Pregnancy complications (hyperemesis, hypertension, and preeclampsia)/Asthma									
Nafstad et al. (28)	2000	questionnaire administered at the maternity wards soon after birth.	4 years	164	2,367	OR	2.5	1.4	4.2
Maternal hypertension/Asthma									
Algert et al. (23)	2011		3 years	7,245	240,511	OR	1,02	0.95	1.11
Yang et al. (22)	2021	Medical records maternal blood pressure at early (≤14 weeks), mid (14–28 weeks), and late (≥28 weeks) pregnancy. Hypertension (SBP ≥ 140 and/or DBP ≥ 90 mm Hg)	3 years	NR	NR	OR	0.94	0,73	1.22
Hypertension before pregnancy/ASTHMA						OR			
Shaheen et al. (5)	2016	General (La hipertensión gestacional se definió como presión arterial sistólica ≥ 140 mmHg y/o presión arterial diastólica ≥ 90 mmHg en dos ocasiones después de 20 semanas de gestación)	7 years	879	7,285	OR	1.34	1.00	1.79
Preeclampsia/recurrent wheezing									
Mirzakhani et al. (18)	2019		3 years	96	NR	HR	4.73	2.20	10.07
Mirzakhani et al. (27)	2019	At least two occasions in two separate years as previously defined, accompanied by a medical diagnosis of asthma at the age of 3 years.	6 years	119	69	RR	1.95	1.13	3.38
Preeclampsia/Wheezing									
Shaheen et al. (5)	2016		18 months	65	158	OR	1.31	0.94	1.82
Shaheen et al. (5)	2016		7 years	18	144	OR	0.98	0.55	1.77
Wilmink et al. (19)	2018	Validated oscillometric digital device (OMRON HEM-907), ISSHP & ACOG criteria	10 years	692	4,202	OR	0.70	0.36	1.39
HPD/Wheezing									
Kelly et al.	2022		12 months	145	1,440	OR	1.26	01.04	1.51
Yang et al. (22)	2021	Medical record review	3–13 years	NR	NR	OR	1.01	0.90	1.13
Gestional hypertension/Wheezing									
Henderson and Quenby (21)	2021	Early wheeze	5 years	3,805		OR	1.22	01.02	1.45
Henderson and Quenby (21)	2021	Severe wheeze	5 years	924		0R	1.18	0.89	1.58
Shaheen et al. (5)	2016		18 months	405	1,154	OR	0.92	0.79	01.06
Shaheen et al. (5)	2016		7 years	134	1,020	OR	01.09	0.87	1.37
Wilmink et al. (19)	2018	Validated oscillometric digital device (OMRON HEM-907), ISSHP & ACOG criteria	10 years	692	4,202	OR	0.95	0.63	2.39
Maternal hypertension/Asthma									
Yang et al. (22)	2021	Medical records maternal blood pressure at early (≤14 weeks), mid (14–28 weeks), and late (≥28 weeks) pregnancy. Hypertension (SBP ≥ 140 and/or DBP ≥ 90 mm Hg)	3 years	NR	NR	OR	0.97	0.80	1.19
Preeclampsia/Uses medication asthma									
Stokholm et al. (24)	2017	General (ICD-8 ICD-10)	until age 7 years	47	289	OR	04.01	1.11	14.13
HDP/Used medication asthma									
Kelly et al.	2022		NR	51	472	OR	1.32	0.98	1.78
Preeclampsia/Lung function (FEV1%)									
Byberg et al. (20)	2014	Mild/moderate	12.8 years	126	NR	b regression coefficient,	0.57	−1.86	2.99
Byberg et al. (20)	2014	Severe pre-eclampsia	12.8 years	45	NR	b regression coefficient,	1.27	−2.95	5.49
Shaheen et al. (5)	2016	General	8 years	NR		MD	−0.14	−0.33	0.06
Wilmink et al. (19)	2018	PE/HELLP	10 years	NR		Z-score	−0.16	−0.38	0.06
Stokholm et al. (24)	2017	General (ICD-8 ICD-10)	until age 7 years	313		b Coefficient	0.17	−0.32	0.67
Preeclampsia/Lung function (FVC)									
Byberg et al. (20)	2014	Mild/moderate	12.8 years	126		b regression coefficient,	−1.47	−4.36	1.42
Byberg et al. (20)	2014	Severe pre-eclampsia	12.8 years	45		b regression coefficient,	−2.24	−7.28	2.79
Shaheen et al. (5)	2016	General	8 years	NR		DM	−0.07	−0.26	0.12
Wilmink et al. (19)	2018	PE/HELLP	10 years	NR		Z-score	−0.10	−0.31	0.11
Preeclampsia/Lung function (FV1%/FVC)									
Byberg et al. (20)	2014	Mild/moderate	12.8 years	126		b regression coefficient,	1.28	−0.35	2.92
Byberg et al. (20)	2014	Severe pre-eclampsia	12.8 years	45		b regression coefficient,	2.79	−0.06	5.64
Wilmink et al. (19)	2018	PE/HELLP	10 years	NR		Z-score	−0.05	−0.27	0.16
Gestional hypertension/Lung function (FEV1%)									
Wilmink et al. (19)	2018	PE/HELLP	10 years	NR		Z-score	0.05	−0.11	0.20
Gestional hypertension/Lung function (FVC)									
Wilmink et al. (19)	2018	PE/HELLP	10 years	NR		Z-score	0.44	−0.10	0.19
Gestional hypertension/ LUNG FUNCTION (FV1%/FVC)									
Wilmink et al. (19)	2018	PE/HELLP	10 years	NR		Z-score	0.02	−0.12	0.17

The synthesis of statistical results revealed variability in the estimated association between HDP and asthma in offspring. Several studies reported a significant positive association, particularly for severe preeclampsia, with odds ratios exceeding 1.20 in multiple analyses. For example, Byberg et al. (14) found that severe preeclampsia was associated with an HR of 1.78 (95% CI: 1.64–1.95) for asthma development in early childhood, while Magnus et al. (15) reported a relative risk of 1.29 (95% CI: 1.19–1.39) for asthma in children born to mothers with preeclampsia. However, some studies did not find a statistically significant association, particularly for mild forms of hypertensive disorders, where confidence intervals overlapped the null value. In addition, studies examining the effect of HDP on other respiratory outcomes, such as recurrent wheezing and lung function, found mixed results, with some suggesting an increased risk and others reporting no significant effect (Table 3).

### Association of hypertensive disorders in pregnancy and risk of asthma in the offspring

**Figure 2:**
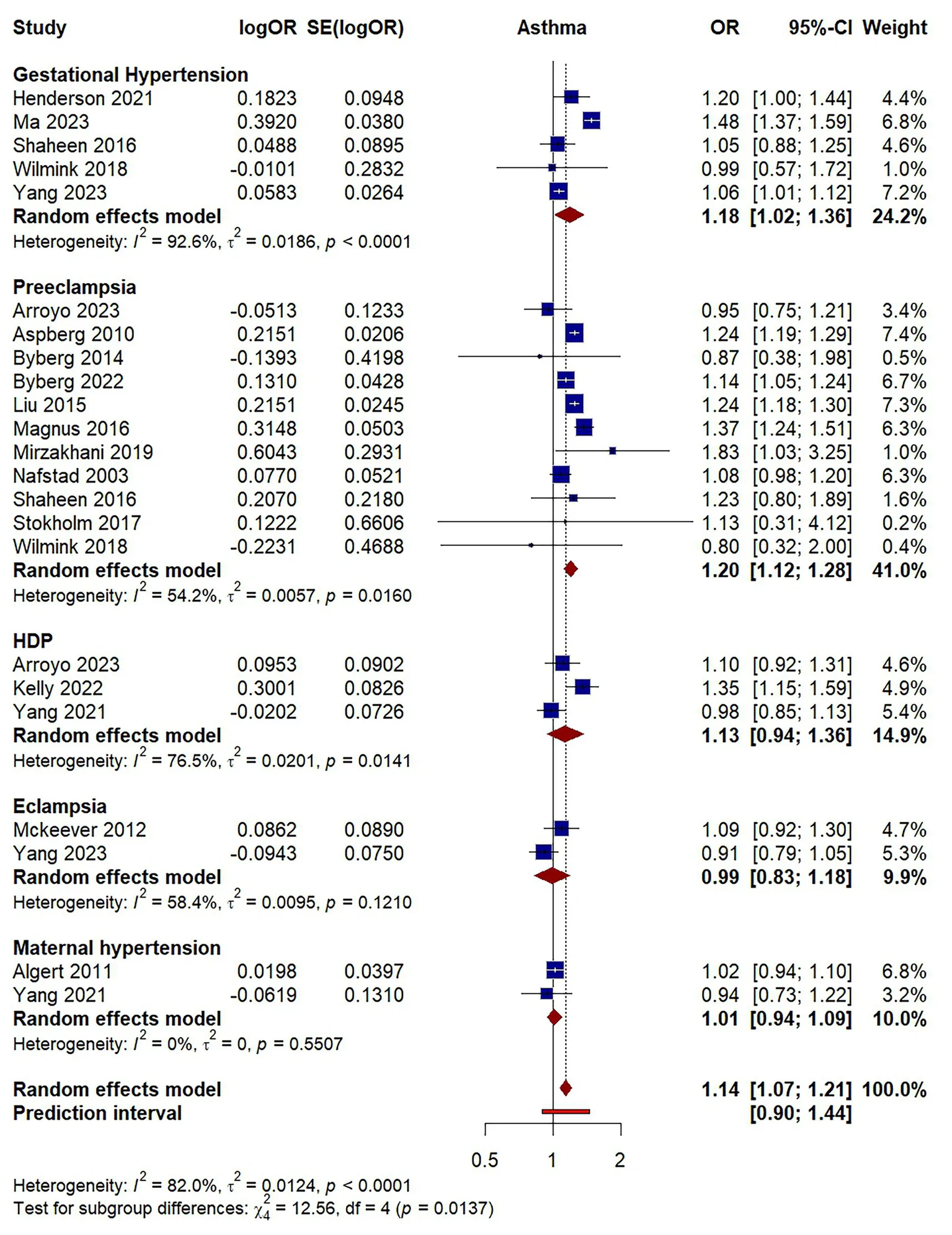
Forest plot of the association between hypertensive disorders of pregnancy and the risk of asthma in the offspring, stratified by HDP subtype (gestational hypertension, preeclampsia, HDP, eclampsia, and maternal hypertension). Pooled estimates were obtained using a random-effects (DerSimonian–Laird) model. OR, odds ratio; 95% CI, 95% confidence interval; *I*^2^, heterogeneity statistic; *τ*^2^, between-study variance.

The meta-analysis evaluating the association between hypertensive disorders of pregnancy (HDP) and the risk of asthma in offspring included 20 studies. The overall pooled estimate, calculated using a random-effects model, was an OR of 1.14 (95% CI: 1.07–1.21), indicating a statistically significant association between HDP and increased risk of childhood asthma. The prediction interval (0.90–1.44) suggests that, although the overall effect is positive, some future studies may not find a significant association. Relevant differences were observed according to the specific type of hypertensive disorder. Gestational hypertension showed a significant association (OR: 1.18; 95% CI: 1.02–1.36; *I*^2^ = 92.6%; *p* < 0.0001), and preeclampsia also presented a positive effect (OR: 1.20; 95% CI: 1.12–1.28; *I*^2^ = 54.2%; *p* = 0.016). For HDP in general, the OR was 1.13 (95% CI: 0.94–1.36; *I*^2^ = 76.5%; *p* = 0.014), without reaching statistical significance. Associations with eclampsia (OR: 0.99; 95% CI: 0.83–1.18; *I*^2^ = 58.4%; *p* = 0.121) and chronic maternal hypertension (OR: 1.01; 95% CI: 0.94–1.09; *I*^2^ = 0%; *p* = 0.551) were not statistically significant. The overall analysis showed high heterogeneity (*I*^2^ = 82.0%; *p* < 0.0001), indicating considerable between-study variability. The test for differences between subgroups was significant (*χ*^2^ = 12.56; *p* = 0.014), reinforcing the need to distinguish between HDP subtypes in future analyses. Studies such as Aspberg (7.4%) (16), Liu (7.3%) (17), Magnus (6.3%) (15), and Byberg (14) (6.7%) (14) contributed greater weight to the pooled model. Some studies, such as Mirzakhani (OR: 1.83) (18) and Kelly (OR: 1.35), (3) suggested stronger associations in certain populations, whereas others, such as Wilmink (OR: 0.80) (19) or Byberg (20) (OR: 0.87) (20), showed no significant effects (Figure 2). Taken together, these findings support a positive association between intrauterine exposure to hypertensive disorders of pregnancy and the risk of asthma in offspring, particularly in cases of gestational hypertension and preeclampsia.

Additional pre-specified subgroup analyses (Supplementary Table 6) showed a positive association between HDP and asthma in offspring across all strata, although effect magnitudes varied modestly. Pooled ORs were similar when stratifying by continent (Europe: OR = 1.16; 95% CI: 1.08–1.25; North America: OR = 1.10; 95% CI: 0.97–1.24; Asia–Pacific: OR = 1.13; 95% CI: 0.99–1.30), by exposed-group sample size (> 500: OR = 1.15; 95% CI: 1.08–1.23; ≤ 500: OR = 1.10; 95% CI: 0.92–1.32), and by year of publication (> 2015: OR = 1.16; 95% CI: 1.08–1.24; ≤ 2015: OR = 1.12; 95% CI: 1.02–1.22). Heterogeneity remained moderate-to-high in most strata, suggesting that residual variability is unlikely to be driven solely by these structural factors and may also reflect differences in case definitions, follow-up duration, and population background.

**Figure 3:**
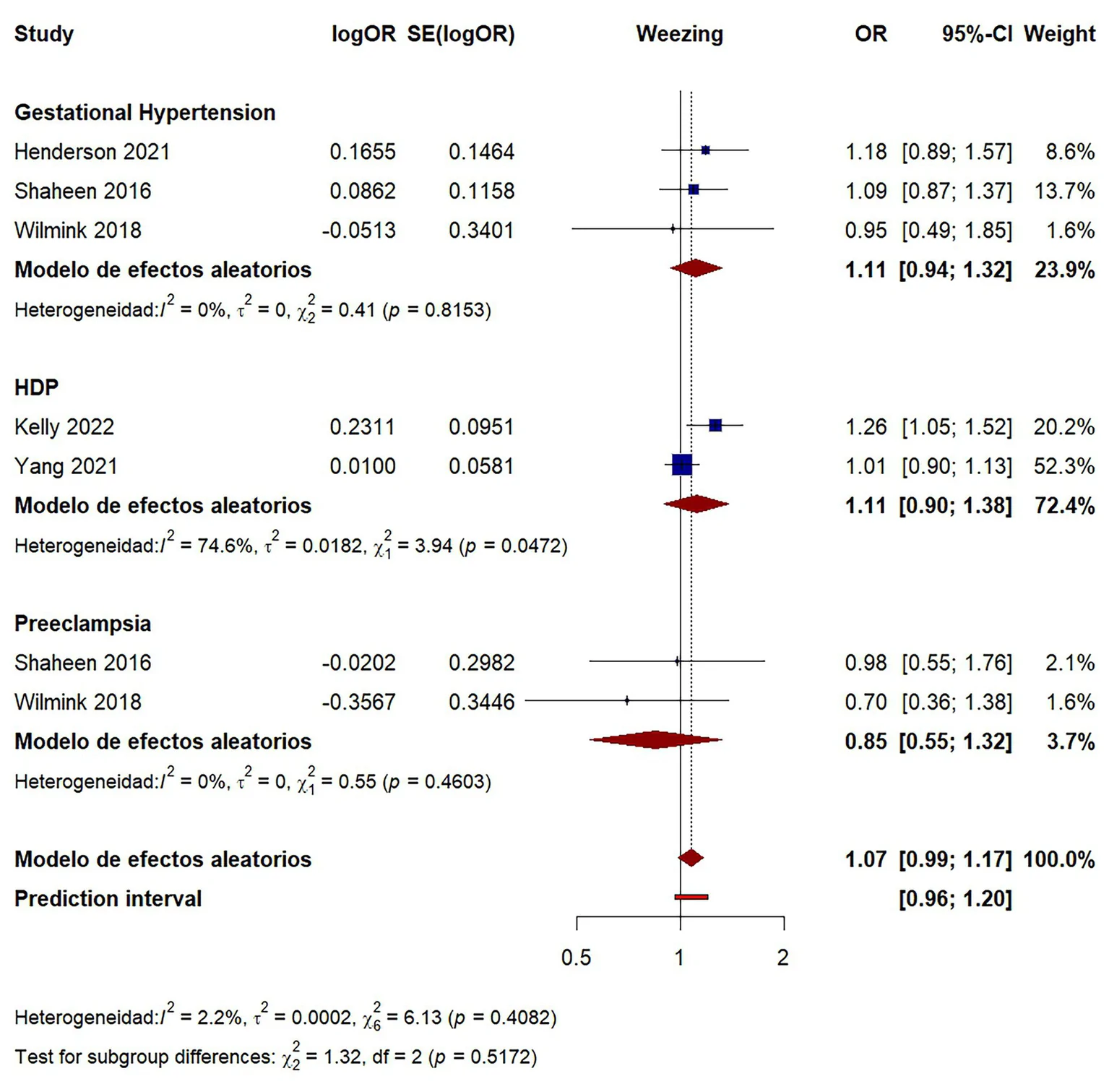
Forest plot of the association between hypertensive disorders of pregnancy and the risk of wheezing in the offspring. Five unique studies provided seven independent effect estimates because two studies (5, 19) contributed separate estimates for gestational hypertension and preeclampsia, displayed as independent rows within their respective subgroups. Pooled estimates were obtained using a random-effects (DerSimonian–Laird) model. OR, odds ratio; 95% CI, 95% confidence interval; *I*^2^, hetero*genei*ty statistic; *τ*^2^, between-study variance.

As a secondary outcome, the association between hypertensive disorders of pregnancy (HDP) and the presence of wheezing in the offspring was evaluated. The meta-analysis included five unique studies (5, 19, 21, 22) contributing seven independent effect estimates, since two studies (5, 19) reported separate estimates for gestational hypertension and preeclampsia and were therefore included in their respective subgroups. The pooled OR was 1.07 (95% CI: 0.99–1.17), without reaching statistical significance. The prediction interval was 0.96–1.20, and overall heterogeneity was low (*I*^2^ = 2.2%; *p* = 0.408). In subgroup analyses, gestational hypertension showed a possible positive trend (OR: 1.11; 95% CI: 0.94–1.32; *I*^2^ = 0%; *p* = 0.815), as did HDP in general (OR: 1.11; 95% CI: 0.90–1.38; *I*^2^ = 74.6%; *p* = 0.047). In contrast, no significant association was observed for preeclampsia (OR: 0.85; 95% CI: 0.55–1.32; *I*^2^ = 0%; *p* = 0.460) (Figure 3).

### Risk of bias assessment

The risk of bias assessment using the Newcastle–Ottawa Scale (NOS) demonstrated an overall low risk of bias across the included studies, with the majority receiving high scores in the selection, comparability, and outcome domains. Most studies achieved the maximum rating in the selection domain, indicating appropriate study design, well-defined exposure criteria, and representative population sampling. The comparability domain, assessing the adequacy of confounder adjustment, showed slight variability, with most studies receiving two stars while a few received only one, suggesting potential residual confounding. The outcome domain, which evaluates the validity and completeness of outcome assessment, was consistently strong across studies, with nearly all scoring the highest possible.

**Figure 4:**
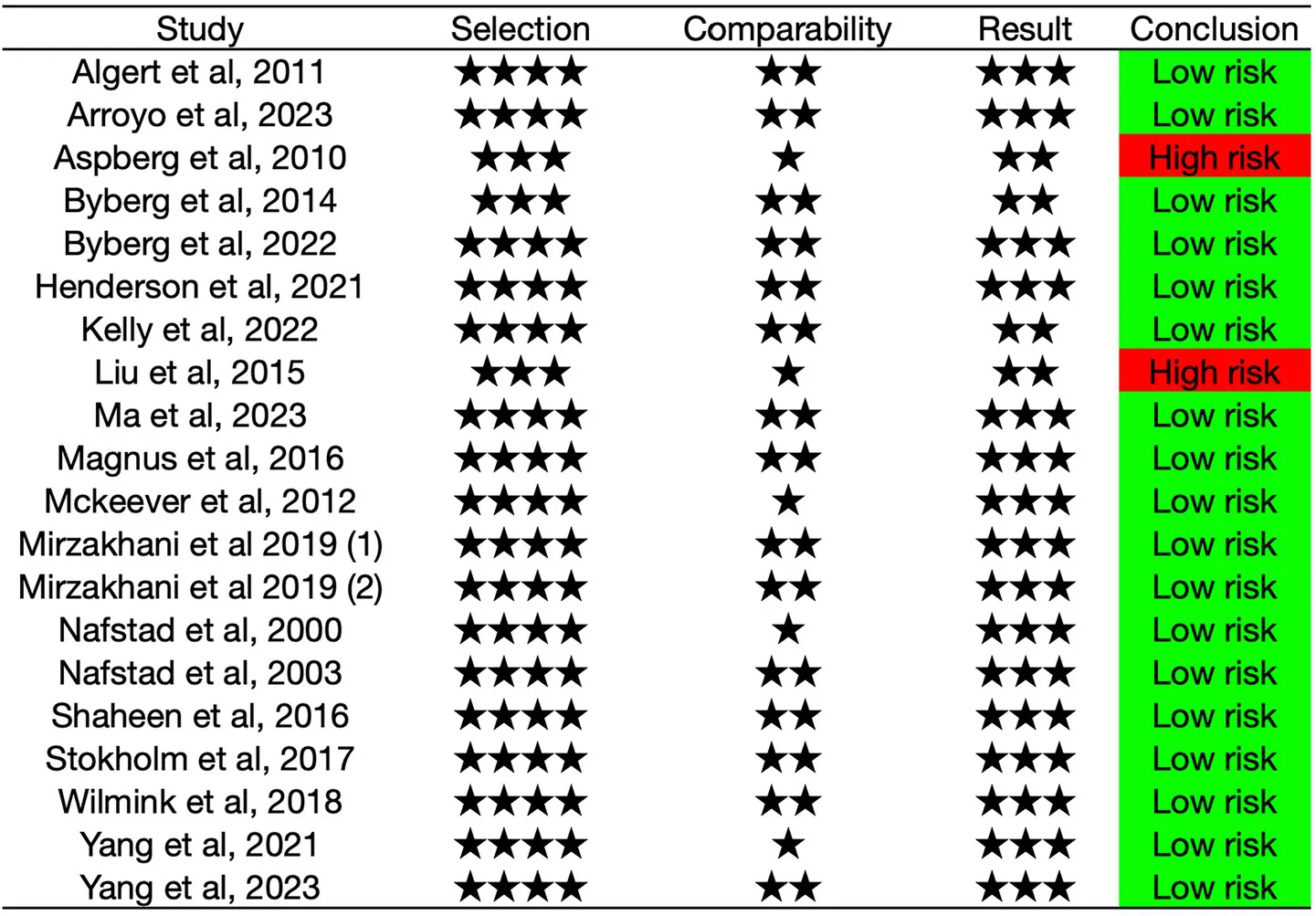
Risk of bias assessment of the included studies according to the Newcastle–Ottawa Scale (selection, comparability, and outcome domains).

Two studies, Ma (4) and Aspberg (16), were classified as high risk due to limitations in comparability and outcome assessment. These studies had either insufficient adjustment for confounders or weaker methodology in asthma diagnosis, which may have impacted the reliability of their effect estimates. However, 80% of the included studies (*n* = 18) (5, 6, 14, 15, 17–29) were generally robust, supporting the reliability of the findings. Most studies demonstrated rigorous methodology and a low likelihood of systematic bias. The conclusions from this risk of bias assessment suggest that the observed association between hypertensive disorders during pregnancy and offspring asthma is unlikely to be significantly influenced by study design limitations, reinforcing the validity of the meta-analysis results (Figure 4).

### Summary of results and certainty of evidence

The overall certainty of the evidence ranged from low to high, depending on the specific exposure and degree of heterogeneity. Five studies evaluated the association between gestational hypertension and asthma, showing a potential increased risk (OR: 1.18; 95% CI: 1.02–1.36), although the certainty of the evidence was rated as low, mainly due to very high inconsistency (*I*^2^ = 92.6%; *p* < 0.0001). For preeclampsia, based on 11 studies, the pooled effect estimate indicated a probable association with asthma in offspring (OR: 1.20; 95% CI: 1.12–1.28). The certainty of the evidence was rated as moderate, with some concern due to heterogeneity (*I*^2^ = 54.2%; *p* = 0.016). For HDP, three studies were included with an OR of 1.13 (95% CI: 0.94–1.36). The certainty of this evidence was also moderate, but the results were not statistically significant and showed substantial inconsistency (*I*^2^ = 76.5%; *p* = 0.014). Regarding eclampsia, the pooled estimate of two studies did not suggest a significant association (OR: 0.99; 95% CI: 0.83–1.18), with moderate certainty of evidence despite moderate heterogeneity (*I*^2^ = 58.4%; *p* = 0.121). Finally, maternal chronic hypertension was evaluated in two studies. The analysis did not indicate an increased risk of asthma (OR: 1.01; 95% CI: 0.94–1.09), and the certainty of the evidence was rated as high, supported by the absence of observed heterogeneity (*I*^2^ = 0%; *p* = 0.551).

## Discussion

Our findings indicate that hypertensive disorders of pregnancy (HDP) are significantly associated with an increased risk of asthma in offspring, supporting the hypothesis that prenatal exposure to an adverse intrauterine environment may contribute to the development of respiratory diseases. The pooled OR of 1.20 (95% CI: 1.12–1.28) for preeclampsia suggests that children born to mothers with this complication have approximately a 20% higher likelihood of developing asthma compared to those born to normotensive pregnancies. These results align with previous systematic reviews and meta-analyses, which have consistently found a moderate but significant association between HDP and atopic diseases, particularly asthma.

The biological plausibility of this association is supported by several mechanisms, including systemic inflammation, placental dysfunction, and oxidative stress, which may lead to altered fetal lung development and immune dysregulation. Chronic placental hypoxia, a hallmark of HDP, has been proposed as a key mediator in fetal programming, contributing to structural and functional abnormalities in the developing airway. Furthermore, maternal hypertension has been linked to increased systemic inflammatory markers and endothelial dysfunction, both of which can impact fetal immune system maturation and predispose offspring to airway hyperresponsiveness.

Beyond asthma, hypertensive disorders of pregnancy have also been associated with other atopic conditions, including eczema and allergic diseases, suggesting the presence of shared underlying mechanisms. One plausible explanation involves intrauterine immune programming, in which maternal inflammation and placental dysfunction may disrupt fetal immune development. In particular, alterations in regulatory T-cell (Treg) function and immune tolerance mechanisms have been proposed as key pathways linking adverse intrauterine environments to increased susceptibility to atopic diseases. These immunological alterations may predispose offspring not only to asthma but to a broader spectrum of allergic conditions, supporting the hypothesis of a common atopic developmental pathway.

In addition, the timing of asthma assessment varied substantially across the included studies and may represent different clinical phenotypes (e.g., transient early wheezing versus persistent school-age or adolescent asthma). Age at outcome assessment is therefore a potential source of heterogeneity and effect modification, since exposure to HDP may have differential effects on the developing immune and respiratory systems depending on the age window in which the outcome is captured.

The results of our meta-analysis are consistent with those of Li et al. (8), who reported an increased risk of asthma in children exposed to preeclampsia in utero, with an overall OR of 1.19 (95% CI: 1.12–1.26). Their findings also demonstrated that the association remained significant after adjusting for potential confounders such as maternal smoking, socioeconomic status, and birth weight, reinforcing the hypothesis that HDP has an independent effect on childhood asthma risk.

Although our findings suggest a robust association, substantial heterogeneity was observed among the included studies (*I*^2^ = 82.0%; *p* < 0.0001), indicating possible differences in study design, exposure definitions, and outcome assessment. One potential explanation is the variation in asthma diagnostic criteria. Some studies relied on physician-diagnosed asthma (6, 14, 15, 17, 19, 22, 24, 25, 29) while others used self-reported parental questionnaires (5, 18, 20, 27), which may have introduced misclassification bias. Additionally, the timing of asthma diagnosis differed across studies, with some assessing outcomes in early childhood and others extending follow-up into adolescence.

Subgroup analyses revealed that the association was stronger in the offspring of mothers with preeclampsia compared with those with gestational hypertension. This finding aligns with Conlan et al. (7), who reported that preeclampsia was associated with a significantly increased risk of asthma (pooled OR: 1.14; 95% CI: 1.04–1.26), whereas the association with other hypertensive disorders of pregnancy was weaker and not statistically significant. These results suggest that the severity of hypertension during pregnancy may influence fetal respiratory outcomes, with preeclampsia exerting a more pronounced adverse effect due to its greater impact on placental function.

Although some individual studies stratified hypertensive disorders by severity (e.g., mild, moderate, and severe preeclampsia), the definitions and classification criteria were not consistent across studies, precluding a robust quantitative synthesis of dose–response effects. Nevertheless, several reports suggest that more severe forms of HDP may be associated with a greater risk of adverse respiratory outcomes, indicating a potential severity-dependent relationship that should be explored in future research using standardized definitions.

Another key finding of our study was the role of publication bias, as suggested by the asymmetry in the funnel plot and confirmed by Egger’s and Begg’s tests. Applying the trim-and-fill method indicated that the observed effect might be slightly overestimated. However, even after adjusting for potential missing studies, the association between HDP and asthma remained statistically significant, indicating that our findings are unlikely to be entirely driven by selective reporting.

The study by Wang et al. (30) further extends our understanding of the long-term respiratory consequences of HDP, demonstrating that maternal hypertension is not only associated with childhood asthma but also with an increased risk of developing chronic obstructive pulmonary disease (COPD) later in life. Their findings highlight the potential long-term implications of intrauterine exposure to HDP, suggesting that maternal hypertension may contribute to lifelong respiratory dysfunction through epigenetic modifications and altered immune system programming.

Despite the strengths of our study, including a rigorous systematic review methodology and the use of meta-analytic techniques to synthesize the evidence, several limitations must be acknowledged. First, the included studies varied in their adjustment for confounders, and residual confounding cannot be ruled out. Factors such as maternal atopy, environmental pollution, and early-life respiratory infections were not consistently accounted for, potentially influencing the observed associations. Second, most studies included in our analysis were from high-income countries, limiting the generalizability of our findings to low- and middle-income settings, where the prevalence of HDP and asthma risk factors may differ. Third, the lack of individual patient-level data precluded a more detailed exploration of effect modification by genetic predisposition, mode of delivery, or neonatal respiratory interventions.

In addition, environmental and postnatal factors known to influence asthma development—such as air pollution, tobacco smoke exposure, early-life respiratory infections, and socioeconomic conditions—were not consistently measured or adjusted for across the included studies. These factors may act as confounders or mediators and could partially explain the observed associations, highlighting the possibility of residual confounding.

In conclusion, our findings provide strong epidemiological evidence supporting an association between HDP and an increased risk of asthma in offspring. The results underscore the importance of maternal cardiovascular health in shaping offspring respiratory outcomes and highlight the need for early-life interventions to mitigate asthma risk in children born to mothers with hypertensive complications. Future research should focus on elucidating the underlying mechanisms, investigating potential gene–environment interactions, and evaluating preventive strategies to improve maternal–fetal health outcomes.

## Data Availability

The datasets presented in this study can be found in online repositories. The names of the repository/repositories and accession number(s) can be found in the article/Supplementary material.
