# Correction: Hypertensive disorders in pregnancy and risk of asthma in the offspring: a systematic review and meta-analysis

**DOI:** 10.3389/fmed.2026.1961705

**Published:** 2026-08-21

**Authors:** Nancy D. Calzada, Carlos A. Barba, Carlos Quispe-Vicuña, Raul H. Sandoval-Ato, Christian A. Rodriguez-Saldaña, Judith Yangali-Vicente, Oriana Rivera-Lozada, Joshuan J. Barboza

**Affiliations:** 1 Universidad de Huánuco, Huánuco, Peru; 2 Hospital d'Olot i comarcal de la Garrotxa, Olot, Spain; 3 Grupo de Investigación NEMECS: Neurociencias, Metabolismo, Efectividad Clínica y Sanitaria, Universidad Científica del Sur, Lima, Peru; 4 Escuela de Medicina Humana, Universidad Antenor Orrego, Piura, Peru; 5 Hospital de la Amistad Perú Corea Santa Rosa II-1, Piura, Peru; 6 Facultad de Responsabilidad Social, Universidad Anahuac, Mexico City, Mexico; 7 Vicerrectorado de Investigación, Universidad Señor de Sipán, Chiclayo, Peru; 8 Escuela de Medicina, Universidad Señor de Sipán, Chiclayo, Peru

**Keywords:** childhood asthma, gestational hypertension, hypertensive disorders of pregnancy, meta-analysis, preeclampsia, systematic review

Affiliation “Tau-Relaped Group, Trujillo, Peru” was erroneously included for author “Carlos Quispe-Vicuña”. The correct affiliation is “Grupo de Investigación NEMECS: Neurociencias, Metabolismo, Efectividad Clínica y Sanitaria, Universidad Científica del Sur, Lima, Peru.”

Affiliation “Hospital III-1 EsSalud José Cayetano Heredia, Piura, Peru” was erroneously included for author “Christian A. Rodriguez-Saldaña”. The correct affiliation is “Hospital de la Amistad Perú Corea Santa Rosa II-1, Piura, Peru.” Author Christian A. Rodriguez-Saldaña is affiliated with affiliation 4 and “Hospital de la Amistad Perú Corea Santa Rosa II-1, Piura, Peru.”

The original version of this article has been updated.

