# Supplementary material for: Hypertensive disorders in pregnancy and risk of asthma in the offspring: a systematic review and meta-analysis
Source: Front Med (Lausanne). 2026 Jul 28;13:1739697. doi: 10.3389/fmed.2026.1739697 (PMC13459099; doi:10.3389/fmed.2026.1739697)
Supplement: Supplementary file 1 [file Supplementary_File_1.docx]

**Hypertensive disorders in pregnancy and risk of asthma in the offspring**: **a systematic review** **and meta-analysis**

**SUPPLEMENTARY MATERIAL**

**Supplemental Table 1. PRISMA 2020 Checklist**

| Section and Topic | Item # | Checklist item | Location where item is reported (N° page) |
| --- | --- | --- | --- |
| TITLE | | |  |
| Title | 1 | Identify the report as a systematic review. | 1 |
| ABSTRACT | | |  |
| Abstract | 2 | See the PRISMA 2020 for Abstracts checklist. | 2 |
| INTRODUCTION | | |  |
| Rationale | 3 | Describe the rationale for the review in the context of existing knowledge. | 4 |
| Objectives | 4 | Provide an explicit statement of the objective(s) or question(s) the review addresses. | 4 |
| METHODS | | |  |
| Eligibility criteria | 5 | Specify the inclusion and exclusion criteria for the review and how studies were grouped for the syntheses. | 5-6 |
| Information sources | 6 | Specify all databases, registers, websites, organisations, reference lists and other sources searched or consulted to identify studies. Specify the date when each source was last searched or consulted. | 5 |
| Search strategy | 7 | Present the full search strategies for all databases, registers and websites, including any filters and limits used. | 5 |
| Selection process | 8 | Specify the methods used to decide whether a study met the inclusion criteria of the review, including how many reviewers screened each record and each report retrieved, whether they worked independently, and if applicable, details of automation tools used in the process. | 6-7 |
| Data collection process | 9 | Specify the methods used to collect data from reports, including how many reviewers collected data from each report, whether they worked independently, any processes for obtaining or confirming data from study investigators, and if applicable, details of automation tools used in the process. | 6-7 |
| Data items | 10a | List and define all outcomes for which data were sought. Specify whether all results that were compatible with each outcome domain in each study were sought (e.g. for all measures, time points, analyses), and if not, the methods used to decide which results to collect. | 7 |
|  | 10b | List and define all other variables for which data were sought (e.g. participant and intervention characteristics, funding sources). Describe any assumptions made about any missing or unclear information. | 7 |
| Study risk of bias assessment | 11 | Specify the methods used to assess risk of bias in the included studies, including details of the tool(s) used, how many reviewers assessed each study and whether they worked independently, and if applicable, details of automation tools used in the process. | 7 |
| Effect measures | 12 | Specify for each outcome the effect measure(s) (e.g. risk ratio, mean difference) used in the synthesis or presentation of results. | 7 |
| Synthesis methods | 13a | Describe the processes used to decide which studies were eligible for each synthesis (e.g. tabulating the study intervention characteristics and comparing against the planned groups for each synthesis (item #5)). | 7 |
|  | 13b | Describe any methods required to prepare the data for presentation or synthesis, such as handling of missing summary statistics, or data conversions. | 7 |
|  | 13c | Describe any methods used to tabulate or visually display results of individual studies and syntheses. | 7 |
|  | 13d | Describe any methods used to synthesize results and provide a rationale for the choice(s). If meta-analysis was performed, describe the model(s), method(s) to identify the presence and extent of statistical heterogeneity, and software package(s) used. | 7 |
|  | 13e | Describe any methods used to explore possible causes of heterogeneity among study results (e.g. subgroup analysis, meta-regression). | 8 |
|  | 13f | Describe any sensitivity analyses conducted to assess robustness of the synthesized results. | 7 |
| Reporting bias assessment | 14 | Describe any methods used to assess risk of bias due to missing results in a synthesis (arising from reporting biases). | 7 |
| Certainty assessment | 15 | Describe any methods used to assess certainty (or confidence) in the body of evidence for an outcome. | 7-8 |
| RESULTS | | |  |
| Study selection | 16a | Describe the results of the search and selection process, from the number of records identified in the search to the number of studies included in the review, ideally using a flow diagram. | 9 |
|  | 16b | Cite studies that might appear to meet the inclusion criteria, but which were excluded, and explain why they were excluded. | 9 |
| Study characteristics | 17 | Cite each included study and present its characteristics. | 9 |
| Risk of bias in studies | 18 | Present assessments of risk of bias for each included study. | 9-10 |
| Results of individual studies | 19 | For all outcomes, present, for each study: (a) summary statistics for each group (where appropriate) and (b) an effect estimate and its precision (e.g. confidence/credible interval), ideally using structured tables or plots. | 10, Table 1 and Table 2 |
| Results of syntheses | 20a | For each synthesis, briefly summarise the characteristics and risk of bias among contributing studies. | 11 |
|  | 20b | Present results of all statistical syntheses conducted. If meta-analysis was done, present for each the summary estimate and its precision (e.g. confidence/credible interval) and measures of statistical heterogeneity. If comparing groups, describe the direction of the effect. | 11-12 |
|  | 20c | Present results of all investigations of possible causes of heterogeneity among study results. | 12 |
|  | 20d | Present results of all sensitivity analyses conducted to assess the robustness of the synthesized results. | 11-12 |
| Reporting biases | 21 | Present assessments of risk of bias due to missing results (arising from reporting biases) for each synthesis assessed. | 12 |
| Certainty of evidence | 22 | Present assessments of certainty (or confidence) in the body of evidence for each outcome assessed. | 11 |
| DISCUSSION | | |  |
| Discussion | 23a | Provide a general interpretation of the results in the context of other evidence. | 14 |
|  | 23b | Discuss any limitations of the evidence included in the review. | 16-17 |
|  | 23c | Discuss any limitations of the review processes used. | 16-17 |
|  | 23d | Discuss implications of the results for practice, policy, and future research. | 15-16 |
| OTHER INFORMATION | | |  |
| Registration and protocol | 24a | Provide registration information for the review, including register name and registration number, or state that the review was not registered. | 5 |
|  | 24b | Indicate where the review protocol can be accessed, or state that a protocol was not prepared. | 5 |
|  | 24c | Describe and explain any amendments to information provided at registration or in the protocol. | 5 |
| Support | 25 | Describe sources of financial or non-financial support for the review, and the role of the funders or sponsors in the review. | 1 |
| Competing interests | 26 | Declare any competing interests of review authors. | 1 |
| Availability of data, code and other materials | 27 | Report which of the following are publicly available and where they can be found: template data collection forms; data extracted from included studies; data used for all analyses; analytic code; any other materials used in the review. | Supplemental Tables |

*From:*  Page MJ, McKenzie JE, Bossuyt PM, Boutron I, Hoffmann TC, Mulrow CD, et al. The PRISMA 2020 statement: an updated guideline for reporting systematic reviews. BMJ 2021;372:n71. doi: 10.1136/bmj.n71. This work is licensed under CC BY 4.0. To view a copy of this license, visit<https://creativecommons.org/licenses/by/4.0/>

**Supplemental Table 2. Search strategy on each databases**

| **Database** | **Search strategy**  **Search date:** December 3, 2024 | | **Results** |
| --- | --- | --- | --- |
| **Pubmed** | #1 | "Hypertension, Pregnancy Induced" OR "Pregnancy-Induced Hypertension" OR "Gestational Hypertension" OR "Hypertension, Gestational" OR "Pregnancy Induced Hypertension" OR "Hypertensions, Pregnancy Induced" OR "Induced Hypertension, Pregnancy" OR "Induced Hypertensions, Pregnancy" OR "Transient Hypertension, Pregnancy" OR "Hypertension, Pregnancy Transient" OR "Pregnancy Transient Hypertension" OR Eclampsia OR Eclampsias OR "HELLP Syndrome" OR "Syndrome, HELLP" OR "Hemolysis, Elevated Liver Enzymes, Lowered Platelets" OR "Pre Eclampsia" OR "Preeclampsia" OR "Pregnancy Toxemias" OR "Pregnancy Toxemia" OR "Toxemia, Pregnancy" OR "Edema-Proteinuria-Hypertension Gestosis" OR "Edema Proteinuria Hypertension Gestosis" OR "Gestosis, Edema-Proteinuria-Hypertension" OR "Hypertension-Edema-Proteinuria Gestosis" OR "Gestosis, Hypertension-Edema-Proteinuria" OR "Hypertension Edema Proteinuria Gestosis" OR "Proteinuria-Edema-Hypertension Gestosis" OR "Gestosis, Proteinuria-Edema-Hypertension" OR "Proteinuria Edema Hypertension Gestosis" OR "EPH Complex" OR "EPH Toxemias" OR "EPH Toxemia" OR "Toxemia, EPH" OR "Toxemias, EPH" OR "EPH Gestosis" OR "Gestosis, EPH" OR "Toxemias, Pregnancy" OR "Toxemia Of Pregnancy" OR "Of Pregnancies, Toxemia" OR "Of Pregnancy, Toxemia" OR "Pregnancies, Toxemia Of" OR "Pregnancy, Toxemia Of" OR "Toxemia Of Pregnancies" OR "Preeclampsia Eclampsia 1" OR "1, Preeclampsia Eclampsia" OR "1s, Preeclampsia Eclampsia" OR "Eclampsia 1, Preeclampsia" OR "Eclampsia 1s, Preeclampsia" OR "Preeclampsia Eclampsia 1s" | 362 |
|  | #2 | "Asthma" OR "Asthmas" OR "Asthma, Bronchial" OR "Bronchial Asthma" |  |
|  | #3 | #1 AND #2 |  |
| **WoS** | #1 | "Hypertension, Pregnancy Induced" OR "Pregnancy-Induced Hypertension" OR "Gestational Hypertension" OR "Hypertension, Gestational" OR "Pregnancy Induced Hypertension" OR "Hypertensions, Pregnancy Induced" OR "Induced Hypertension, Pregnancy" OR "Induced Hypertensions, Pregnancy" OR "Transient Hypertension, Pregnancy" OR "Hypertension, Pregnancy Transient" OR "Pregnancy Transient Hypertension" OR Eclampsia OR Eclampsias OR "HELLP Syndrome" OR "Syndrome, HELLP" OR "Hemolysis, Elevated Liver Enzymes, Lowered Platelets" OR "Pre Eclampsia" OR "Preeclampsia" OR "Pregnancy Toxemias" OR "Pregnancy Toxemia" OR "Toxemia, Pregnancy" OR "Edema-Proteinuria-Hypertension Gestosis" OR "Edema Proteinuria Hypertension Gestosis" OR "Gestosis, Edema-Proteinuria-Hypertension" OR "Hypertension-Edema-Proteinuria Gestosis" OR "Gestosis, Hypertension-Edema-Proteinuria" OR "Hypertension Edema Proteinuria Gestosis" OR "Proteinuria-Edema-Hypertension Gestosis" OR "Gestosis, Proteinuria-Edema-Hypertension" OR "Proteinuria Edema Hypertension Gestosis" OR "EPH Complex" OR "EPH Toxemias" OR "EPH Toxemia" OR "Toxemia, EPH" OR "Toxemias, EPH" OR "EPH Gestosis" OR "Gestosis, EPH" OR "Toxemias, Pregnancy" OR "Toxemia Of Pregnancy" OR "Of Pregnancies, Toxemia" OR "Of Pregnancy, Toxemia" OR "Pregnancies, Toxemia Of" OR "Pregnancy, Toxemia Of" OR "Toxemia Of Pregnancies" OR "Preeclampsia Eclampsia 1" OR "1, Preeclampsia Eclampsia" OR "1s, Preeclampsia Eclampsia" OR "Eclampsia 1, Preeclampsia" OR "Eclampsia 1s, Preeclampsia" OR "Preeclampsia Eclampsia 1s" | 290 |
|  | #2 | "Asthma" OR "Asthmas" OR "Asthma, Bronchial" OR "Bronchial Asthma") |  |
|  | #3 | #1 AND #2 |  |
| **Scopus** | #1 | TITLE-ABS-KEY("Hypertension, Pregnancy Induced" OR "Pregnancy-Induced Hypertension" OR "Gestational Hypertension" OR "Hypertension, Gestational" OR "Pregnancy Induced Hypertension" OR "Hypertensions, Pregnancy Induced" OR "Induced Hypertension, Pregnancy" OR "Induced Hypertensions, Pregnancy" OR "Transient Hypertension, Pregnancy" OR "Hypertension, Pregnancy Transient" OR "Pregnancy Transient Hypertension" OR Eclampsia OR Eclampsias OR "HELLP Syndrome" OR "Syndrome, HELLP" OR "Hemolysis, Elevated Liver Enzymes, Lowered Platelets" OR "Pre Eclampsia" OR "Preeclampsia" OR "Pregnancy Toxemias" OR "Pregnancy Toxemia" OR "Toxemia, Pregnancy" OR "Edema-Proteinuria-Hypertension Gestosis" OR "Edema Proteinuria Hypertension Gestosis" OR "Gestosis, Edema-Proteinuria-Hypertension" OR "Hypertension-Edema-Proteinuria Gestosis" OR "Gestosis, Hypertension-Edema-Proteinuria" OR "Hypertension Edema Proteinuria Gestosis" OR "Proteinuria-Edema-Hypertension Gestosis" OR "Gestosis, Proteinuria-Edema-Hypertension" OR "Proteinuria Edema Hypertension Gestosis" OR "EPH Complex" OR "EPH Toxemias" OR "EPH Toxemia" OR "Toxemia, EPH" OR "Toxemias, EPH" OR "EPH Gestosis" OR "Gestosis, EPH" OR "Toxemias, Pregnancy" OR "Toxemia Of Pregnancy" OR "Of Pregnancies, Toxemia" OR "Of Pregnancy, Toxemia" OR "Pregnancies, Toxemia Of" OR "Pregnancy, Toxemia Of" OR "Toxemia Of Pregnancies" OR "Preeclampsia Eclampsia 1" OR "1, Preeclampsia Eclampsia" OR "1s, Preeclampsia Eclampsia" OR "Eclampsia 1, Preeclampsia" OR "Eclampsia 1s, Preeclampsia" OR "Preeclampsia Eclampsia 1s") | 282 |
|  | #2 | TITLE-ABS-KEY("Asthma" OR "Asthmas" OR "Asthma, Bronchial" OR "Bronchial Asthma") |  |
|  | #3 | #1 AND #2 |  |
| **Embase** | #1 | ('maternal hypertension' | 476 |
|  | #2 | 'asthma' |  |
|  | #3 | #1 AND #2 |  |

**Suplemental Table 3.** Summary of Findings for hypertensive disorders in pregnancy and risk of asthma in the offspring: a systematic review and meta-analysis

| **Outcomes  ( follow -up)** | **Number of studies** | **Measure of effect** | **Certainty** | **Reasons for reduction** | **Interpretation** |
| --- | --- | --- | --- | --- | --- |
| Gestational Hypertension | 5 | OR 1.18 (1.02–1.36) | ⨁⨁◯◯  Low ^a^ | Inconsistency Very serious (I²=92.6%) | Gestational hypertension may increase the risk of asthma, but certainty is low. |
| Preeclampsia | 11 | OR 1.20 (1.13–1.27) | ⨁⨁⨁ ◯  Moderate ^b^ | Inconsistency (I²=50.4%) | Preeclampsia is probably associated with an increased risk of asthma in offspring. The certainty is moderate. |
| HDP | 3 | OR 1.13 (0.94–1.36) | ⨁⨁⨁ ◯  Moderate ^b^ | Inconsistency (I²=76.5%) | Hypertensive disorders in pregnancy probably do not show a clear association with the risk of asthma in offspring. The evidence is moderate. |
| Eclampsia | 2 | OR 0.99 (0.83–1.18) | ⨁⨁⨁ ◯  Moderate ^b^ | Inconsistency (I²=58.4%) | Eclampsia is probably not significantly associated with the risk of asthma in offspring. The certainty is moderate. |
| Maternal Hypertension | 2 | OR 1.01 (0.94–1.09) | ⨁⨁⨁⨁  High | Inconsistency (I²=0.0%) | Maternal hypertension during pregnancy does not appear to affect the risk of asthma in offspring. The evidence is high certainty. |

**Explanations of certainty of evidence:**

a. Certainty was downgraded by two levels due to inconsistency, as the I² > 80%.
b. Certainty was downgraded by one level due to inconsistency, as the I² is between 40% and 80%.

**Supplemental Table 4. List of studies that were assessed in full-text and excluded.**

| **Author and year** | **Title** | **Exclusion reason** |
| --- | --- | --- |
| Arroyo AC. (2022) | Maternal hypertensive disorders of pregnancy and the risk of childhood asthma | No full text |
| Kelly L. (2019) | An association between hypertensive disorders in pregnancy and childhood diagnosis of asthma | No full text |
| Kelly L. (2019) | Hypertensive disorders in pregnancy and childhood diagnosis of asthma | No full text |
| Nachum S. (2020) | In utero exposure to pre‐eclampsia as an independent risk factor for long‐term respiratory disease | Different outcome |
| Pesce G. (2019) | Fetal Exposure to Maternal Pregnancy Complications and Respiratory Health in Childhood | Measure of effect without numerical |
| Wang YX. (2023) | Hypertensive disorders of pregnancy and risk of asthma and chronic obstructive pulmonary disease: a prospective cohort study | Different outcome population |
| Zhang G. (2019) | Maternal blood pressure throughout pregnancy, gestational hypertension, preeclampsia and offspring asthma. | Conference abstract |
| Zhang G. (2019) | Concomitance of maternal asthma and preeclampsia and asthma risk in the offspring. | Conference abstract |

# **Supplemental Table 5**. Comparison with previous systematic reviews and meta-analyses

| **Study** | **Years included** | **Number of Studies** | **Quality tools** | **Result** | **I² (%)** | **Key Findings** | **Meta-analysis** | **Studies included in the meta-analysis** |
| --- | --- | --- | --- | --- | --- | --- | --- | --- |
| Conlan et al. (2021) | April, 2020 | 09 | Quality assessment tool described by McDonald et al.  GRADE  Funnel plot | Preeclampsia/Asthma | 62% | 1.14 (1.04, 1.26) | **Preeclampsia with asthma**  Mirzakhani, 2019; Wilmick, 2018; Stokhholm, 2017  Magnus, 2016; Shaheen, 2016; Byberg, 2014  Nafstad, 2003; McKeever, 2002 | - McKeever - ( Exposure = Eclampsia) - Studies were not included: Aspberg, 2010 and Liu, 2015 |
|  |  |  |  | HDP/Asthma | 0% | 1.02 (0.96, 1.09) | **HDP with asthma**  Wilmik, 2018; Pesce, 2017  Shaheen, 2016; Algert, 2011 | - Wilmik 's , 2018 (Exposure = Gestational hypertension and preeclampsia - Pesce, 2017 (Measurement of effect without numerical (figure) - Shaheen, 2016 (Exposure = Gestational hypertension and preeclampsia) - Algert, 2011 (Exposure =Maternal hypertension) |
| Li et al. (2021) | January 2020 | 10 | Newcastle-Ottawa Scale (NOS)  Funnel plot  Egger test  Begg's test  Trim -and - fill procedure | Hypertensive disorders in pregnancy | 72.5 | 1.19 (1.12, 1.26) | Mirzakhani, 2019  Wilmick, 2018  Stockholm, 2017  Shaheen, 2016  Magnus, 2016  Liu, 2015  Byberg, 2014  Aspberg, 2010  Rusconi, 2007  Nafstad , 2003 | - Rusconi, outcome evaluated weezing), - Forest plot, with different classifications of Hypertensive disorders in pregnancy) (preeclampsia, Gestational hypertension, HDP, hypertension before pregnancy) |
| Present study (2025) | Until 2024 | 17 | Newcastle-Ottawa Scale (NOS)  Funnel plot  GRADE  SOF Table | Gestational hypertension / asthma | 92.6% | 1.18(1.02;1.36) | Henderson, 2021  Tue, 2023  Shaheen, 2016  Wilmik , 2018  Yang, 2023 |  |
|  |  |  |  | Preeclampsia/ Asthma | 50.4% | 1-20 (1.13; 1.27) | Arroyo, 2023  Aspberg , 2010  Byberg, 2014  Byberg, 2022  Liu, 2015  Magnus, 2016  Mirzakhani, 2019  Nafstad , 2003  Shaheen, 2016  Stockholm, 2017  Wilmink , 2018 |  |
|  |  |  |  | HDP/ Asthma | 76.5% | 1.13 (0.94;1.36) | Arroyo, 2023  Kelly, 2022  Yang, 2021 |  |
|  |  |  |  | Eclampsia/ **Asthma** | 58.4% | 0.99 (0.83;1.18) | McKeever , 2012  Yang, 2023 |  |
|  |  |  |  | Maternal hypertension / **Asthma** | 0% | 1.01 (0.94;1.09) | Algert , 2011  Yang, 2021 |  |
|  |  |  |  | Gestational hypertension / **Weezing** | 1.11 | 1.11(0.94;1.32) | Henderson, 2021  Shaheen , 2016  Wilmink , 2018 |  |
|  |  |  |  | HDP/ **Weezing** | 1.11 | 1.11 (0.90:1.38) | Kelly, 2022  Yang, 2021 |  |
|  |  |  |  | Preeclampsia/ **Weezing** | 0.85 | 0.85 (0.55;1.32) | Saheheen , 2016  Wilmink , 2018 |  |

# **Supplemental Table 6. Additional pre-specified subgroup analyses of the association between hypertensive disorders of pregnancy and asthma in the offspring**

| **Subgrouping variable** | **Stratum** | **Pooled OR (95% CI)** |
| --- | --- | --- |
| Continent | Europe | 1.16 (1.08–1.25) |
|  | North America | 1.10 (0.97–1.24) |
|  | Asia–Pacific | 1.13 (0.99–1.30) |
| Exposed-group sample size | > 500 | 1.15 (1.08–1.23) |
|  | ≤ 500 | 1.10 (0.92–1.32) |
| Year of publication | > 2015 | 1.16 (1.08–1.24) |
|  | ≤ 2015 | 1.12 (1.02–1.22) |

All estimates are pooled odds ratios (OR) with 95% confidence intervals (CI) derived from random-effects models. All subgroup analyses were pre-specified. Heterogeneity (I²) remained moderate to high across most strata. A positive association between HDP and asthma in the offspring was observed across all strata, with modest variation in effect magnitude. Values correspond to those reported in the main text.
